# Chemical Kinetics Investigations of Dibutyl Ether Isomers Oxidation in a Laminar Flow Reactor

**DOI:** 10.1021/acs.energyfuels.4c03432

**Published:** 2024-10-31

**Authors:** Nimal Naser, Samah Y. Mohamed, Gina M. Fioroni, Seonah Kim, Robert L. McCormick

**Affiliations:** † National Renewable Energy Laboratory, Golden, Colorado 80401, United States; ‡ Chemistry Department, Colorado State University, Fort Collins, Colorado 80523, United States

## Abstract

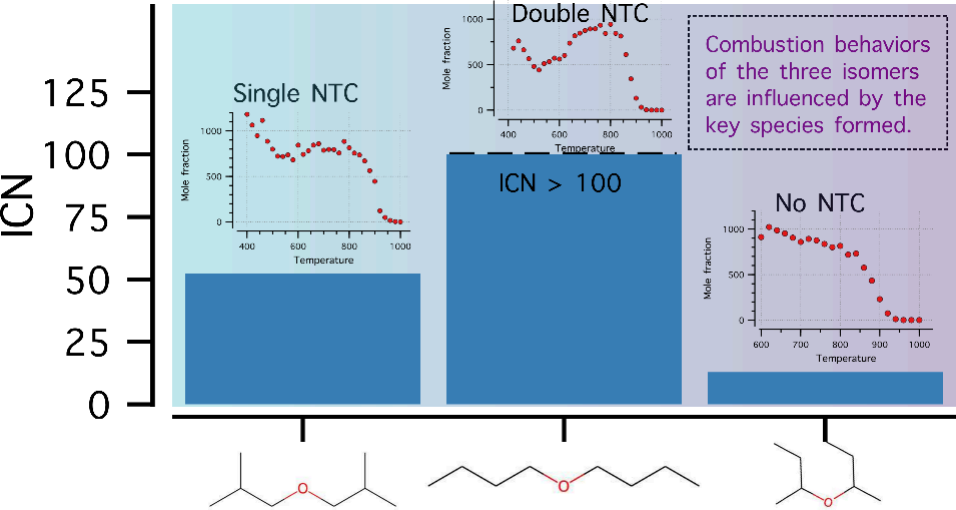

The combustion kinetics of three symmetric diesel-boiling-range ether isomers were investigated experimentally using a plug flow reactor. The isomers were di-*n*-butyl ether (DNBE), diisobutyl ether (DIBE), and di-*sec*-butyl ether (DSBE). The flow reactor experiments employed oxygen as the oxidizer and helium as the diluent, with oxidation carried out at atmospheric and elevated pressure conditions and temperatures from 400 to 1000 at 20 K intervals. The fuel, oxidizer, and diluent flow rates were varied at different temperatures to maintain a constant initial fuel mole fraction of 1000 ppm under stoichiometric conditions and a residence time of 2 s. Reaction products were analyzed by gas chromatography (GC). Depending on the structure, ethers showed different degrees of negative temperature coefficient (NTC) behavior. Speciation results from the GC analysis were then compared to simulations using existing and newly developed chemical kinetic models. Most of the simulated product concentrations showed reasonable agreement with the experimental data. The chemical kinetic models were utilized to elucidate key features of the reactivity and NTC behavior of the different isomers. The chemical kinetic analysis indicates that the combustion behaviors of the three isomers are influenced by the key species formed at the low-temperature reaction regime. The key species identified for DNBE, DIBE, and DSBE at atmospheric pressure are *n*-butanal, isobutanal, and *sec*-butanol, respectively.

## 1 Introduction

*The U.S. National Blueprint for Transportation Decarbonization* presents a plan for eliminating nearly all greenhouse gas emissions by 2050 from the transportation sector.^1^ The plan presents a different strategy for each transportation mode, with light-duty, medium-duty, short-haul, heavy-duty, and buses largely being electrified. Long-haul trucks would operate on a mix of battery-electric, fuel cell, and combustion engines fueled by high-energy-density liquid fuels with low net carbon emissions.^2^ The study suggests that while combustion-engine-powered vehicles would make up 20% of the fleet, they would consume 50% of the energy used in this sector in 2050. Today, low-carbon fuels for diesel engines that are produced from fats and oils are available at commercial scale (biodiesel and renewable diesel).^3^ However, fats and oils feedstocks are a limited resource,^4^ and other resources such as woody biomass and wet waste must be utilized to increase the production of low-carbon-intensity fuels for diesel engines.

While there are many pathways to produce diesel fuels or blend components from biomass,^5^ several studies show that diesel-boiling-range monoethers (with boiling point (BP) nominally 175 to 300 °C) have good potential for reasonable costs, high energy content, and low carbon intensity. The freezing point is typically very low, and the flash point is adequately high, but the cetane number (CN) and soot formation tendency can vary widely with molecular structure.^6^ For example, Fioroni et al. screened a large number of potential diesel blend components and identified di-*n*-pentyl ether based on its BP of 190 °C, flash point of 57 °C, CN over 100, and yield sooting index (YSI)^7^ of only 44.^8^ In a subsequent engine combustion study,^9^ a similar ether reduced nitrogen oxides (NO_*x*_) and soot emissions, with no impact on thermal efficiency. Huq et al. designed the C_11_ ether 4-butoxyheptane based on predicted properties and showed that it could be produced from fermentation-derived butyric acid. This ether boils at 198 °C and has a flash point of 68 °C, CN of 80, and YSI of 58.^10^ Eagan et al. used Guerbet coupling to convert ethanol into C_4_ to C_8_ alcohols that were dehydrated to produce a mixture of C_8_ to C_16_ ethers.^11^ While the mixed product was not characterized, the components boiled in the diesel range and had a very low melting point, high flash point, CN over 100, and YSI well below 100. If produced from cellulosic ethanol, life cycle greenhouse gas emissions were more than 50% lower than petroleum diesel,^12^ and a surrogate mixture for this fuel was successfully used in an engine combustion study.^13^

However, a systematic understanding of the combustion characteristics of ethers is lacking in the literature. While some kinetics studies have been conducted, limited experimental data hinder chemical kinetic model development of ethers for transport fuel applications.^14^ To begin to address fundamental questions about how ether molecular structure impacts combustion, we studied the combustion of three symmetric isomers of dibutyl ether (DBE). While the butyl ethers boil slightly outside of the diesel range, these smaller molecules were chosen because they are experimentally and computationally easier to handle. The three ethers studied are shown in Table 1, along with measured fuel properties. These isomers cover a remarkable range of CN, from 13 to over 100, likely representing several orders of magnitude difference in reactivity.

**Table 1:**
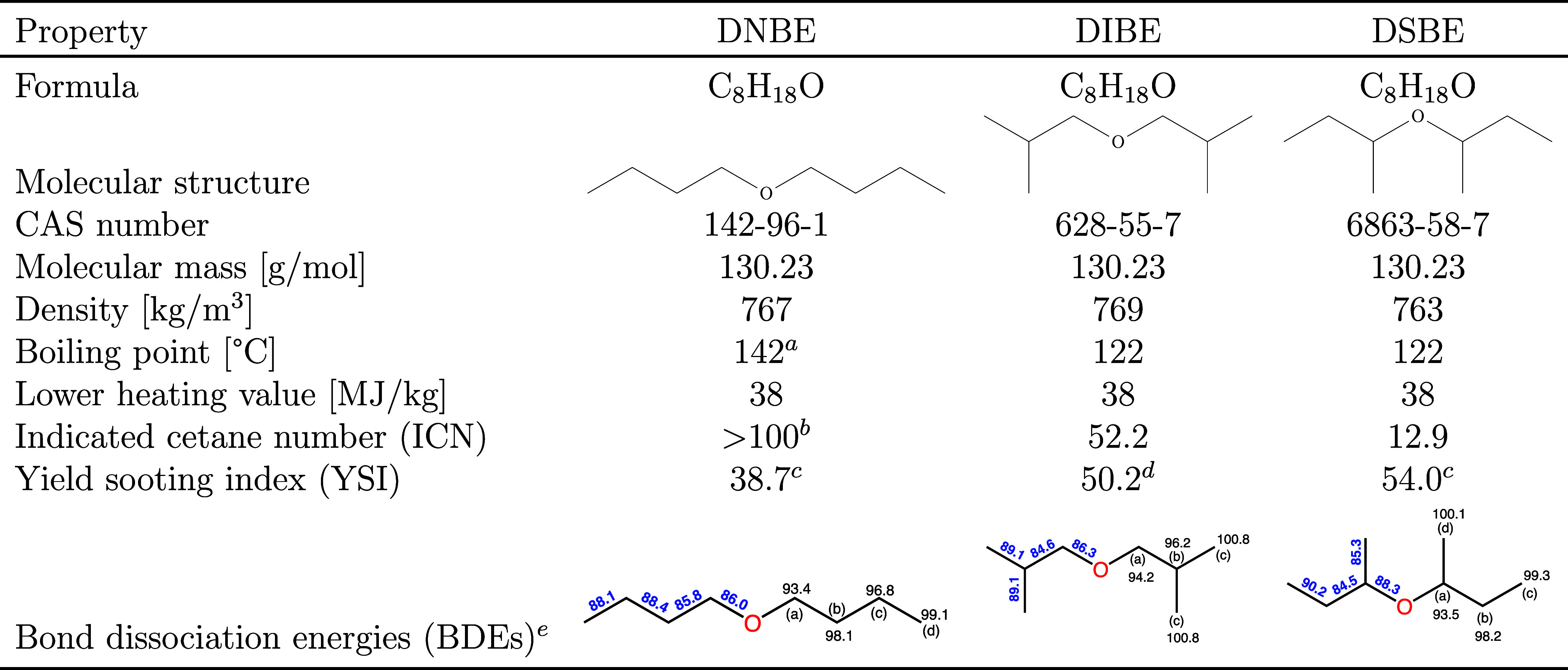
Fuel Properties of the DBE Isomers

a Measured value obtained from Eagen et al.^11^

b ICN over 100 is not defined, and the IDT is shorter than *n*-hexadecane (ICN = 100) indicating higher ICN.

c Measured YSI from McEnally et al.^21^

d Estimated YSI from ml.nrel.gov/.^7,22^

e Letters denote radical sites, and values in blue and black indicate BDE in kcal/mol for C–C (or C–O) and C–H bonds, respectively.

Cai et al.^15^ developed the first chemical kinetic model for di-*n*-butyl ether (DNBE) oxidation with appropriate low-temperature chemical pathways. The model was validated with ignition delay times (IDTs) obtained from a laminar flow reactor and laminar flame speed obtained from a stagnation flame configuration^16^ with satisfactory results. The IDTs from the laminar flow reactor were obtained from the time required to observe a temperature increase when the mixture autoignites, for a predetermined preheat temperature and mass flow rates in the reactor. Their model was able to elucidate several DNBE oxidation pathways; in the low-temperature regime, the keto-hdyroperoxide (KHP) dissociation that leads to radical chain branching was the dominant pathway for DNBE ignition. Thion et al.^17^ experimentally investigated the autoignition behavior of DNBE in a jet-stirred reactor (JSR) and developed a detailed chemical kinetic model with base chemistry developed by Fenard et al.^18^ and Cai et al. models.^15^ Thion et al.^17^ observed two negative temperature coefficient (NTC) regions in the JSR measurements with DNBE at 10 atm. Tran et al.^19^ investigated the oxidation chemistry of DNBE at the low-temperature range at stoichiometric conditions with a plug flow reactor. Similar observations of two NTC regions in the oxidation of DNBE were reported in Tran et al.^19^ With the aid of sophisticated detection techniques, new stable and reactive intermediates during DNBE autoignition were revealed. Based on the newly detected species, they proposed governing chemistries to explain the observed behavior of the two NTC regions. They suggested that the competition between low-temperature chain branching and chain propagation (β-scission or cyclic ether formation) of QOOH radicals is responsible for the first NTC, while the competition between initiating low-temperature chemistry (R + O_2_ ⇌ RO_2_) and the β-scission of the fuel radical is the reason for the second NTC region. Zhong and Han^20^ studied the oxidation of DNBE in a shock tube (ST) and rapid compression machine (RCM) and developed a modified model based on Thion et al.’s model^17^ and were able to predict the NTC behavior in ST/RCM experiments. Zhong and Han’s model is the most up to date in the literature and serves as the starting model for our investigations that are discussed in the next section.

In this work, we consider three isomers of DBE: DNBE, diisobutyl ether (DIBE), and di-*sec*-butyl ether (DSBE). The combustion chemistry of these isomers will be compared using an existing model for DNBE^20^ and new models for DIBE and DSBE developed as part of this work. The models were validated against flow reactor experimental data. Detailed analysis will be performed to enable the understanding of the effects of branching and its position on ether’s oxidation characteristics. Important fuel properties of the DBE isomers are listed in Table 1.

## 2 Methodology

### 2.1 Experiments

A laminar flow reactor was utilized to obtain the autoignition characteristics of the three structural isomers of DBE at two different pressures: atmospheric and elevated pressures of 10 bar. The atmospheric pressure at the National Renewable Energy Laboratory in Golden, Colorado, was 0.84 bar but will be referred to as 1 bar through the rest of the text. The atmospheric and elevated pressure experimental speciation profiles were obtained from two experimental setups. The laminar flow reactor used for 1 bar study was a quartz tube with an internal diameter of 26 mm and length of 73.7 cm. The laminar flow reactor was placed in a furnace with a heated length of 71.1 cm. For the atmospheric flow reactor, a syringe pump was used for fuel introduction directly into the hot zone of the reactor, thereby reducing the reactor length for the residence time calculations. The effective length, i.e., the length from the fuel syringe tip to the outlet end of the reactor, was 62.2 cm. The elevated pressure flow reactor had a nearly identical setup with two differences: (1) the quartz tube was replaced with a SilcoTek oxygen-resistant coated steel tube, and (2) a fuel syringe pump delivery system injecting fuel into a mixing chamber placed upstream of the reactor inlet, and as a result the reactor length for the residence time calculations was 71.1 cm. Experiments at both pressures were carried out at stoichiometric conditions at a residence time of approximately 2 s with oxygen and helium as the oxidizer and diluent, respectively. The inlet fuel mole fraction for all of the fuels and temperature was fixed at 1000 ppm. For both setups, a metered quantity of fuel based on the equivalence ratio and inlet mole fraction was fed to the reactor with the aid of the fuel syringe pump. The reacted gas from the flow reactor was fed into two separate gas chromatography (GC) systems to detect the product species and for quantitation. The mixing chamber temperature in the elevated pressure system was maintained at 470 K. The sampling line between the reactor and the GC was also maintained at 470 K to prevent condensation of the products. The GC loop was maintained at 520 K. Details on both the experimental setups are available in our earlier works.^23,24^Schematics of the experimental setups, the influent flow rates, the measured reactor temperature profiles, and the speciation data are available in the Supporting Information.

The first GC system, GC1, uses a DB-1 60 m × 320 μm × 1 μm capillary column to separate the higher-carbon-number (>C_5_) reaction products, and the column effluent splits to two different detectors in parallel. These are a flame ionization detector (FID) equipped with a Polyarc methanizer for quantitation and a mass spectrometer (MS) for species identification. The Polyarc detector converts all organic compounds to methane after chromatographic separation to achieve a wider linear range than that from the FID and avoids the need for a large number of calibration standards. The Polyarc is calibrated by introducing liquid *n*-heptane into the flow reactor via a syringe pump at 200 °C with helium as the dilution gas. All other species concentrations are calculated based on carbon number against *n*-heptane using the effective carbon number method.^25^ The second GC system, GC2, utilizes three detectors and is set to detect lighter compounds (C_1_–C_5_). GC2 contains an FID for quantitation of hydrocarbon species and two thermal conductivity detectors (TCDs) for quantitation of CO and CO_2_. GC2 is calibrated using gas standards of known concentrations. The TCDs are calibrated with a separate gas mixture containing 500 ppm each of CO and CO_2_. Quantitation of the species is performed by direct comparison to its respective analyte. Uncertainty analysis was applied to the quantified species profiles with linear error propagation theory. The uncertainty in different parameters/properties used in the calculations are provided in Table S1. The average uncertainty in the quantified species is estimated to be 15%.

### 2.2 Theoretical Calculations

The bond dissociation energy (BDE) of the three DBE isomers and the rate constants of DIBE β-scission reaction were calculated in this work using Gaussian 16 software.^26^ The minimum energy structures for all reactants, products, and transition states were located through conformational analysis at the B3LYP/6-31g(2df,p) level of theory, where all C–O and C–C bonds (except terminal C–CH_3_) were simultaneously rotated by 120°, resulting in 3*^n^* conformers, where *n* is the number of rotors. The minimum energy structures were further optimized at B3LYP/6-311G(2df,2pd) and energies were refined using G4. The enthalpy of formation was then calculated by using isodesmic reactions. The rate constants for DIBE scissions were estimated using similar procedure and the transition state theory in Chemrate software. Arrhenius parameters for these reactions are provided in the Supporting Information.

### 2.3 Chemical Kinetic Models

To simulate the data obtained from the flow reactor, we employed Zhong and Han’s model^20^ for DNBE, which is an updated version of Thion et al.’s model.^17^ Zhong and Han^20^ updated the base chemistry using Aramco 3.0,^27^ the H-abstraction reactions, and the key low-temperature reactions to improve agreement with the experimental IDT data.

We developed semidetailed kinetic models for DIBE and DSBE using the Reaction Mechanism Generator (RMG).^28,29^ RMG is an automated tool that utilizes thermodynamic and reaction databases to generate detailed kinetic models based on user-defined parameters.^28,30^ The thermochemical and kinetic libraries were defined based on those used in the methyl propyl ether, RMG, kinetic model development.^31^ For data not available in the defined libraries, the Benson group additivity method and the RMG training database were used. The initial RMG models were refined by updating the base chemistry using Aramco 3.0,^27^ ensuring that all three DBE models have the same core mechanism. Additionally, the generated RMG models initially included only key high- and low-temperature pathways; therefore, missing reactions within the low-temperature chemistry classes were added. These included alternative isomerization pathways as well as 5- and 8-membered ring isomerization reactions, where RMG included only the reactions proceeding via 6- and 7-membered ring isomerization. Note that RMG excluded reactions leading to RO and ROOH, such as RO_2_ + XO_2_ (where X is H, H_2_, CH_3_, or R), as they are less competitive with the low-temperature RO_2_ isomerization initiation pathway. H-abstractions by OH radical for DIBE and DSBE were updated in analogy to methyl propyl ether^31^ and *sec*-butanol^32^ chemistry, respectively. The β-scission reactions of DIBE were updated using rate constants calculated in this work, while analogous rate constants from DNBE^17^ and methyl propyl ether^31^ chemistry were applied to DIBE. Furthermore, key low-temperature reactions for both DIBE and DSBE were updated, adopting rate constants from literature for the first and second O_2_ addition,^33^ RO_2_ to olefin,^34^ RO_2_ to QOOH,^35^ QOOH to cyclic ether,^36^ and KHP formation.^37^ Key reaction classes of the *sec*-butanol submechanism from Sarathy et al.^32^ were incorporated into the DSBE model, as *sec*-butanol was experimentally observed as an important intermediate at low pressure.

The DBE oxidation reactions were modeled using the developed mechanisms in the plug-flow reactor module in Chemkin-Pro software.^38^ The “fix gas temperature” problem type was used as temperature profiles were provided as simulation inputs. Simulations were conducted at 1 and 10 bar with a constant inlet fuel mole fraction of 1000 ppm at stoichiometric conditions. The volumetric flow rate from the experimental measurements, detailed in the Supporting Information, was applied. Absolute and relative tolerance was reduced to 1.0 × 10^–12^ and 1.0 × 10^–8^, respectively. In cases where convergence was not achieved using the default solver options, “Relaxed Iterations” and “Force Nonnegative Solution” advanced settings were enabled. Mole fractions at the final distance were used as the speciation results, which were plotted against the maximum reactor temperature.

## 3 Results and Discussion

Chemical kinetic modeling was utilized to understand the experimentally observed autoignition chemistry of the DBE isomers in the flow reactor. Initiation reactions such as H-abstraction and unimolecular decomposition of species are significantly influenced by the BDE of the cleaved C–H, C–C, or C–O bonds. Therefore, BDEs are very useful in predicting the initially formed radicals and subsequent pathways. BDEs are also important in understanding the effect of functional groups within the species. The calculated BDEs in this study are depicted in Table 1. Letters designate the position relative to the ether’s functional group, where a–d refer to carbon sites that are α, β, γ, and δ from the ether functional group, respectively (except for DSBE-d, which is β to the ether functional group). Results were compared with ALFABET BDE predictions^39−41^ and G3B3 calculations for DNBE from the literature, as detailed in the Supporting Information. Table 1 shows that the ether functional group lowered the neighboring C–C and C–H BDEs relative to the analogous positions in an alkane, except for the DSBE C–O bond, which is 3.5 kcal/mol more stable than the secondary-tertiary BDE in alkanes.

**Figure 1:**
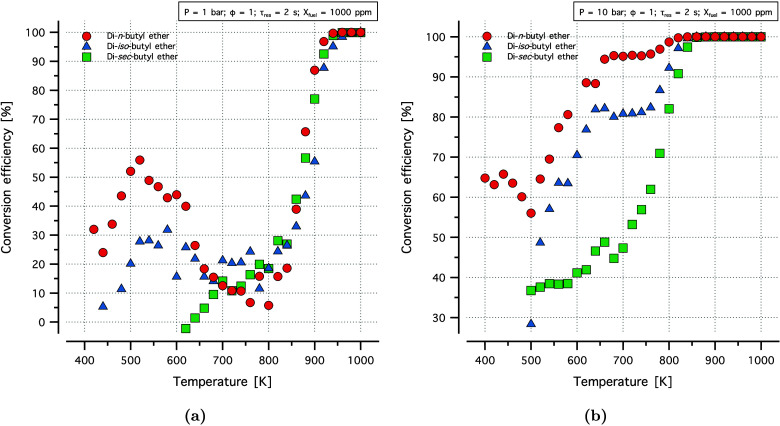
Conversion efficiency of DBE isomers in flow reactor experiments at (a) 1 and (b) 10 bar, stoichiometric conditions, and a residence time of 2 s.

Different DBE isomers exhibited different NTC behavior and reactivity trends. The conversion efficiency () for all three isomers at atmospheric condition and at 10 bar are shown in Figure 1. Based on the conversion efficiency, it can be observed that for both pressure conditions DNBE had the highest conversion at a given temperature and DSBE had the lowest conversion. A comparison of the conversion efficiency and ICN (indicated cetane number) indicates that these values were correlated and that conversion efficiency can be considered as a basis for autoignition reactivity trend assessment. DNBE showed the highest reactivity followed by DIBE and then DSBE. An investigation of the various behaviors observed for each isomer were carried out by flow reactor simulations using the developed models, particularly using sensitivity and flux analysis.

It is important to note that the accuracy of the model predictions is influenced by uncertainties in the thermodynamic data and rate constants that were used, both calculated and measured. The assignment of analogous rate constants is another source of uncertainty. Moreover, missing reaction pathways contribute to these uncertainties, as RMG develops the model based on user-defined parameters, including a threshold for the inclusion of reaction pathways. Additionally, the model is validated against a limited set of experimental flow reactor data, which introduces uncertainty, particularly under conditions that extend beyond those used in the validation experiments.

### 3.1 Di-*n*-butyl Ether

**Figure 2:**
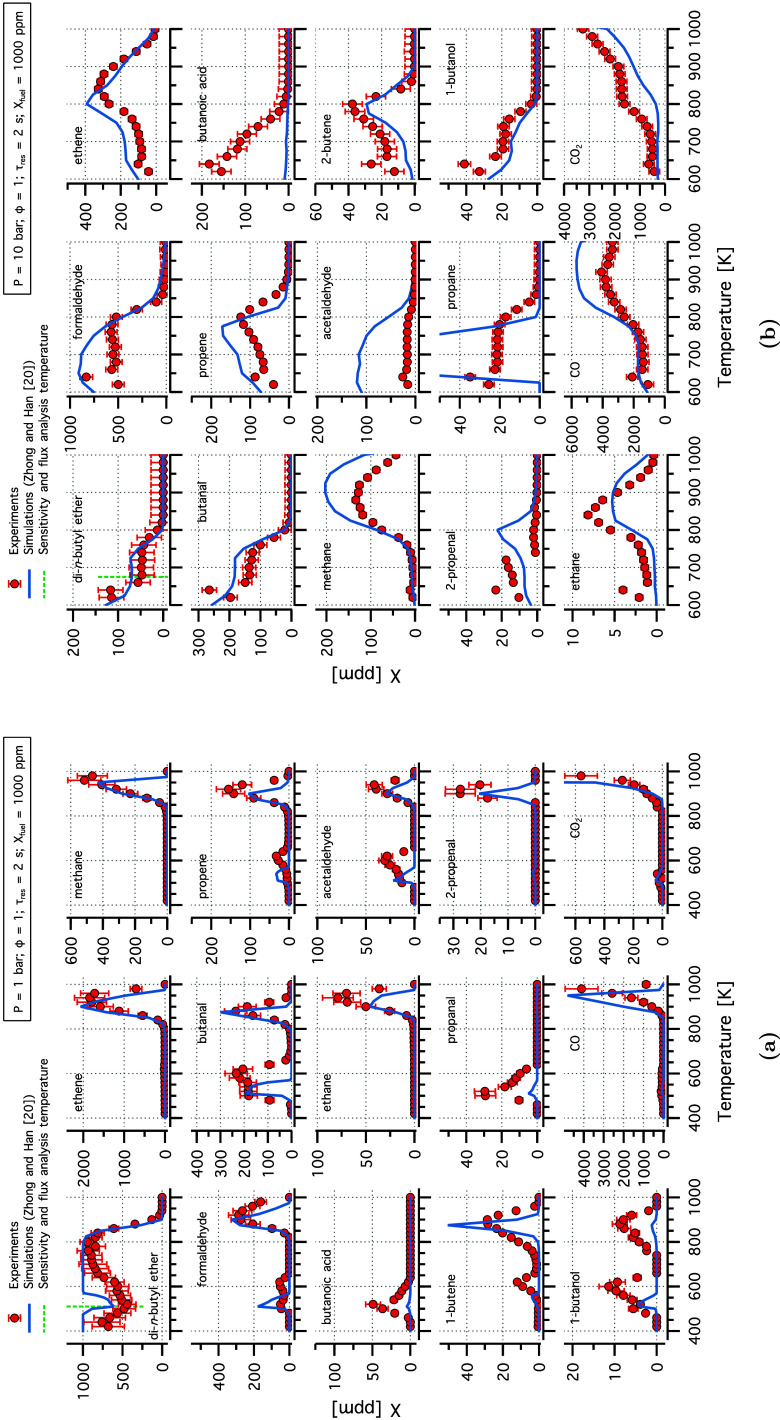
Experimental (symbols) and modeling (lines) mole fraction profiles of species identified in DNBE oxidation in flow reactor experiment at (a) 1 and (b) 10 bar, stoichiometric conditions, and a residence time of 2 s.

Figure 2 shows the experimental speciation results for DNBE at 1 and 10 bar compared to the predictions of the original model by Zhong and Han.^20^ The DNBE experimental data showed a pronounced double NTC behavior at 1 bar (Figure 2a), consistent with observations in the literature.^19^ The model showed good agreement with the experimental observations at 1 bar, particularly for the second peak (800–1000 K). At 10 bar, the model captured the experimental speciation results, except for butanoic acid, acetaldehyde, and propane. In our study, fuel-specific cyclic ethers such as 3-heptyl 1,3-dioxepane, and 3-hydroxypropyl oxirane were detected near the detection limits of the GC. But as sophisticated analytical devices that can accurately differentiate and quantitate these ethers were not available, the scope of the study was directed toward well quantifiable species. Note that several fuel-specific cyclic ethers are formed during oxidation, a detailed quantification of these cyclic ethers was conducted in Tran et al.^19^

**Figure 3:**
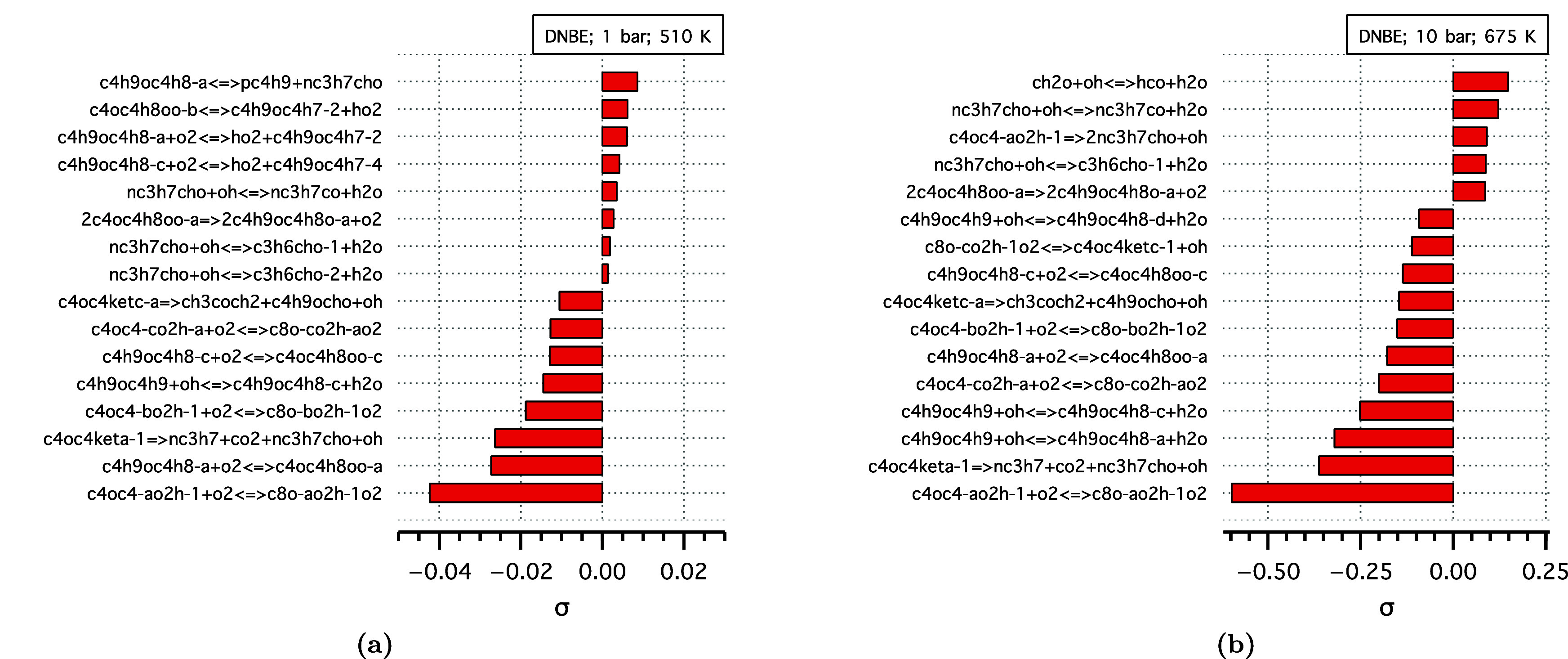
Species sensitivity analysis of DNBE at ϕ = 1 and (a) 1 bar, 510 K, and (b) 10 bar, 675 K.

To determine the governing chemistry and understand the combustion characteristics of DNBE, we performed a sensitivity analysis, as shown in Figure 3. The sensitivity coefficient is defined as σ = ∂ln(*c*_*i*_)/∂ln(*k*_*j*_), where *c*_*i*_ is the concentration of species *i* and *k*_*j*_ is the rate constant of reaction *j*. Negative sensitivities correspond to reactions contributing to fuel consumption, and vice versa. The sensitivity analysis at 1 bar is performed at 510 K, corresponding to the first NTC region. In this region, DNBE consumption is primarily governed by the low-temperature chemistry of the formed radicals, particularly the α-radical to the ether functional group (c4h9oc4h8-a). Positive sensitivity is observed for the competing reaction, including the β-scission of the α-radical and olefin formation (c4h9oc4h7-2 and c4h9oc4h7-4), resulting in the observed NTC behavior. At 10 bar, DNBE consumption is similarly governed by the low-temperature chemistry but with less competition from the QOOH (c4oc4-ao2h-1) β-scission reactions, resulting in relatively higher conversions of DNBE at 10 bar (Figure 2b). Note the contribution of *n*-butanal (nc3h7cho) chemistry to the DNBE sensitivity at both pressures.

**Figure 4:**
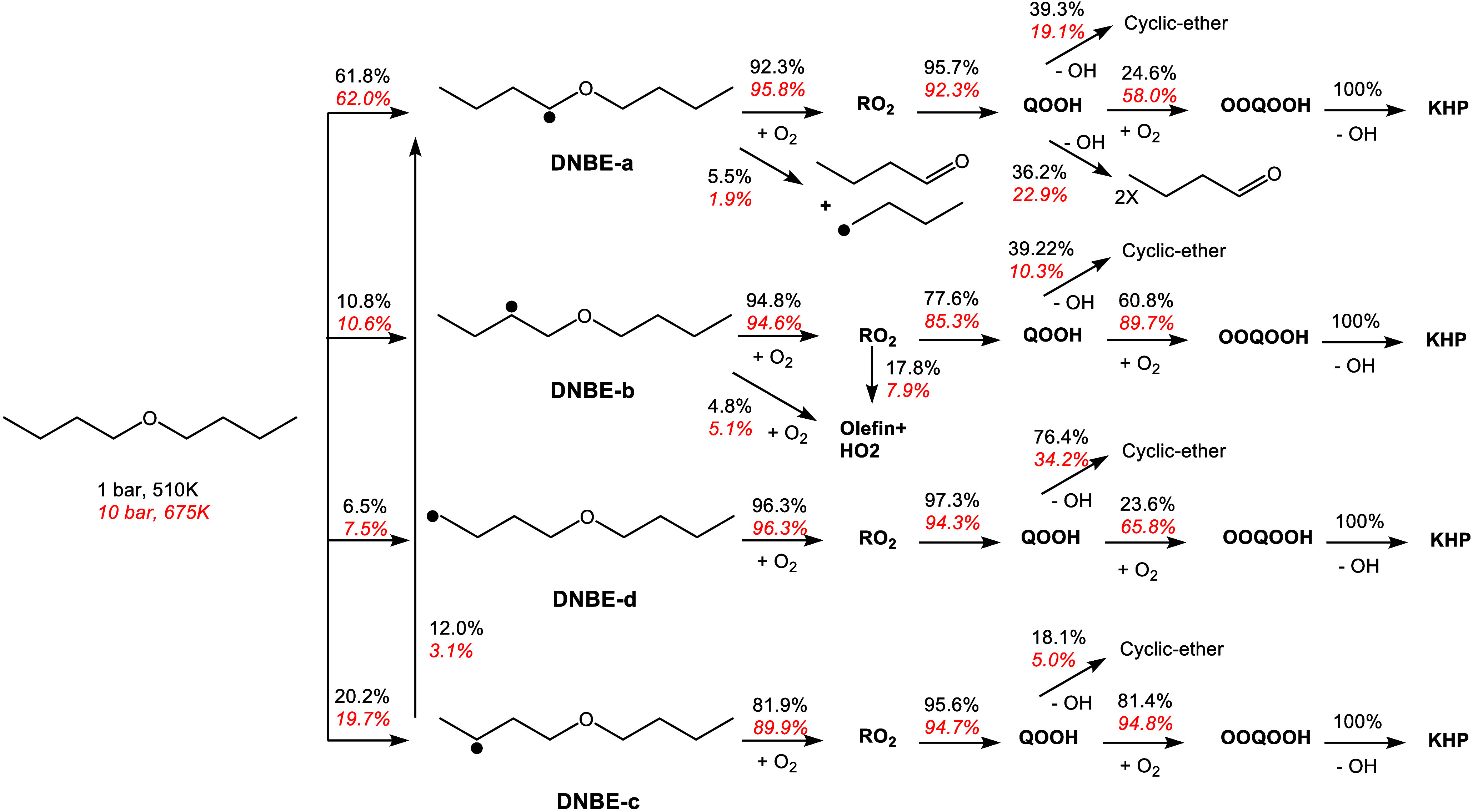
Flux analysis of DNBE at ϕ = 1 and 1 bar, 510 K (black) and 10 bar, 675 K (red, italic).

These sensitivity findings are supported by the flux analysis shown in Figure 4, where approximately 60% of DNBE forms the α-radical (c4h9oc4h8-a), which decomposes via low-temperature reactions via chain branching, chain propagation, or scission of the QOOH radical pathways. The competition among these pathways is responsible for the observed NTC behavior. At high pressure all radicals react via the low-temperature chemistry, where the competition between chain branching (KHP formation) and chain propagation (cyclic ether formation) results in the NTC behavior at 10 bar. It is worth noting that most of the formed DNBE intermediates decompose to form *n*-butanal (nc3h7cho).

### 3.2 Diisobutyl Ether

**Figure 5:**
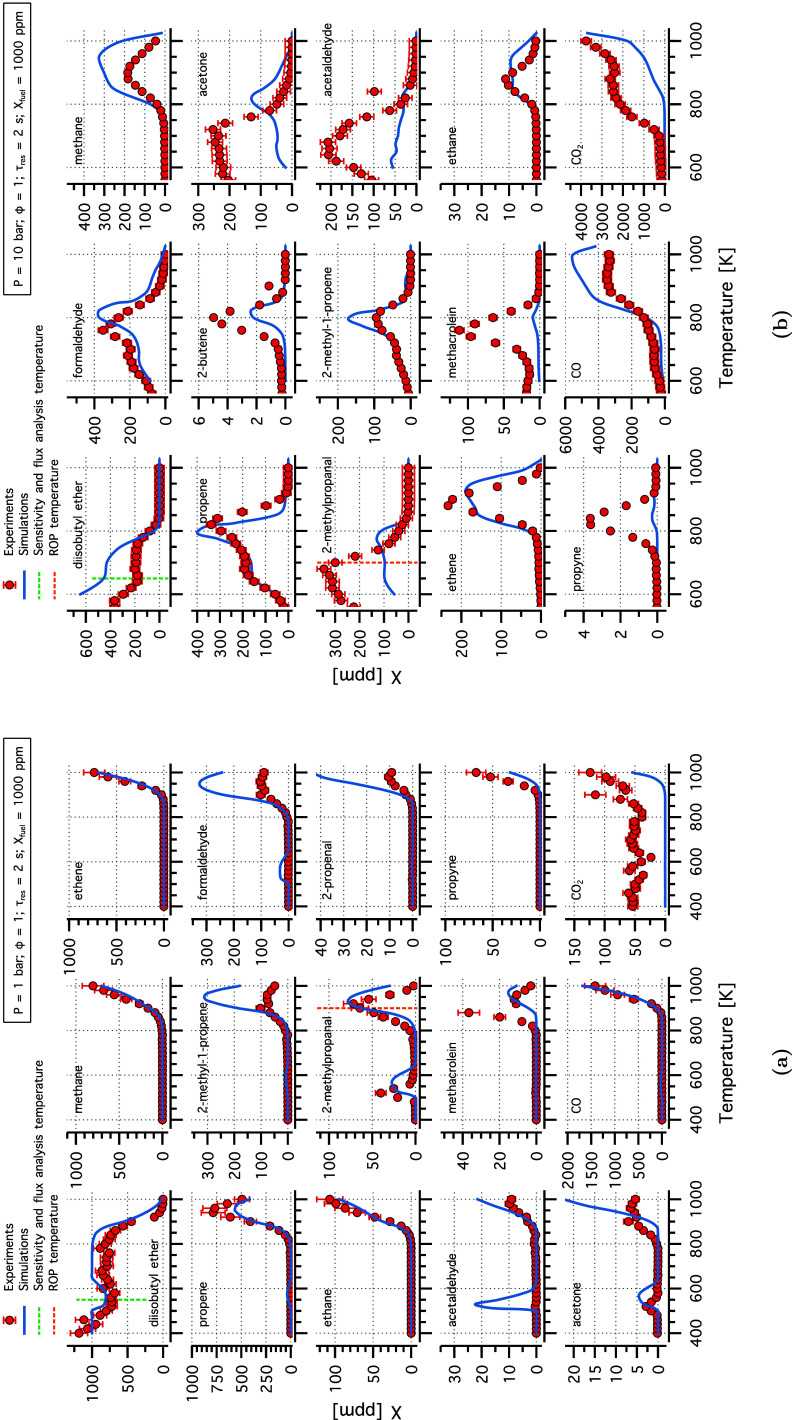
Experimental (symbols) and modeling (lines) mole fraction profiles of species identified in DIBE oxidation in flow reactor experiment at (a) 1 and (b) 10 bar, stoichiometric conditions, and a residence time of 2 s.

The second structural isomer of DBE that was investigated was DIBE, which shows a single NTC region, as observed by the speciation profiles in Figure 5. The simulation agreed well with the experimental data at 1 bar, showing NTC behavior for the fuel and capturing the experimental production rates, especially for key base chemistry species (C_1_–C_3_) and 2-methylpropanal. However, the model overestimated the production of formaldehyde, 2-methyl-1-propene (isobutene), and acetaldehyde, with an additional predicted peak between 500 and 600 K for the latter that was not detected experimentally. At 10 bar, the model qualitatively captured the DIBE consumption profile, showing mild NTC behavior. Good agreement was observed for formaldehyde, isobutene, and the base chemistry species. However, the model underestimated acetone, 2-methylpropanal, and methacrolein, with the peaks shifted by 100 K to higher temperatures. The production of these intermediates is sensitive to the fuel abstraction by OH and the β-scission of QOOH, necessitating more attention computationally to determine site-specific rate constants for this chemistry.

**Figure 6:**
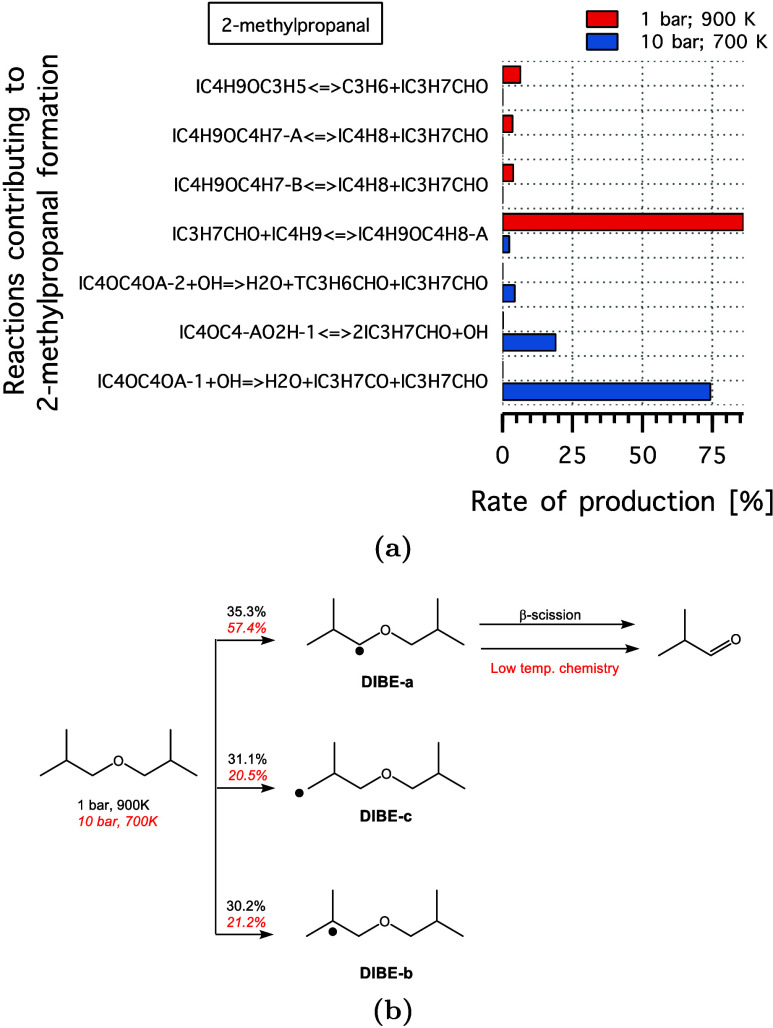
(a) Key reactions contributing to the formation of 2-methylpropanal production from DIBE at 1 bar (900 K) and 10 bar (700 K). (b) Major consumption pathways of DIBE under the same conditions 1 bar (black) and 10 bar (red, italic).

When comparing the experimental speciation data at 1 and 10 bar, both quantitative and qualitative differences are evident. The species peak concentrations at 1 bar are produced at higher temperatures (>800 K) compared to results at 10 bar, even below 600 K for a few species, at 10 bar. This can be attributed to the different chemistry taking place at different pressures. To gain more insight, we employed rate of production (ROP) analysis for 2-methylpropanal, which exhibited a maximum experimental production of 72 and 345 ppm, at 1 bar (900 K) and 10 bar (700 K), respectively, as illustrated in Figure 5. The ROP analysis in Figure 6, showed that at low pressures (and 900 K), 85% of 2-methylpropanal is produced via the β-scission of the α-radical (DIBE-a), where this radical constitutes only 35% of DIBE consumed. However, at higher pressures (and 700 K), the decomposition of low-temperature intermediates accounts for 97% of the produced 2-methylpropanal, particularly cyclic ethers and QOOH radicals formed from DIBE-a, which represents 57% of the fuel. Therefore, the production of 2-methylpropanal at elevated pressure (and 700 K) is expected to occur at lower temperatures with higher rates of production.

**Figure 7:**
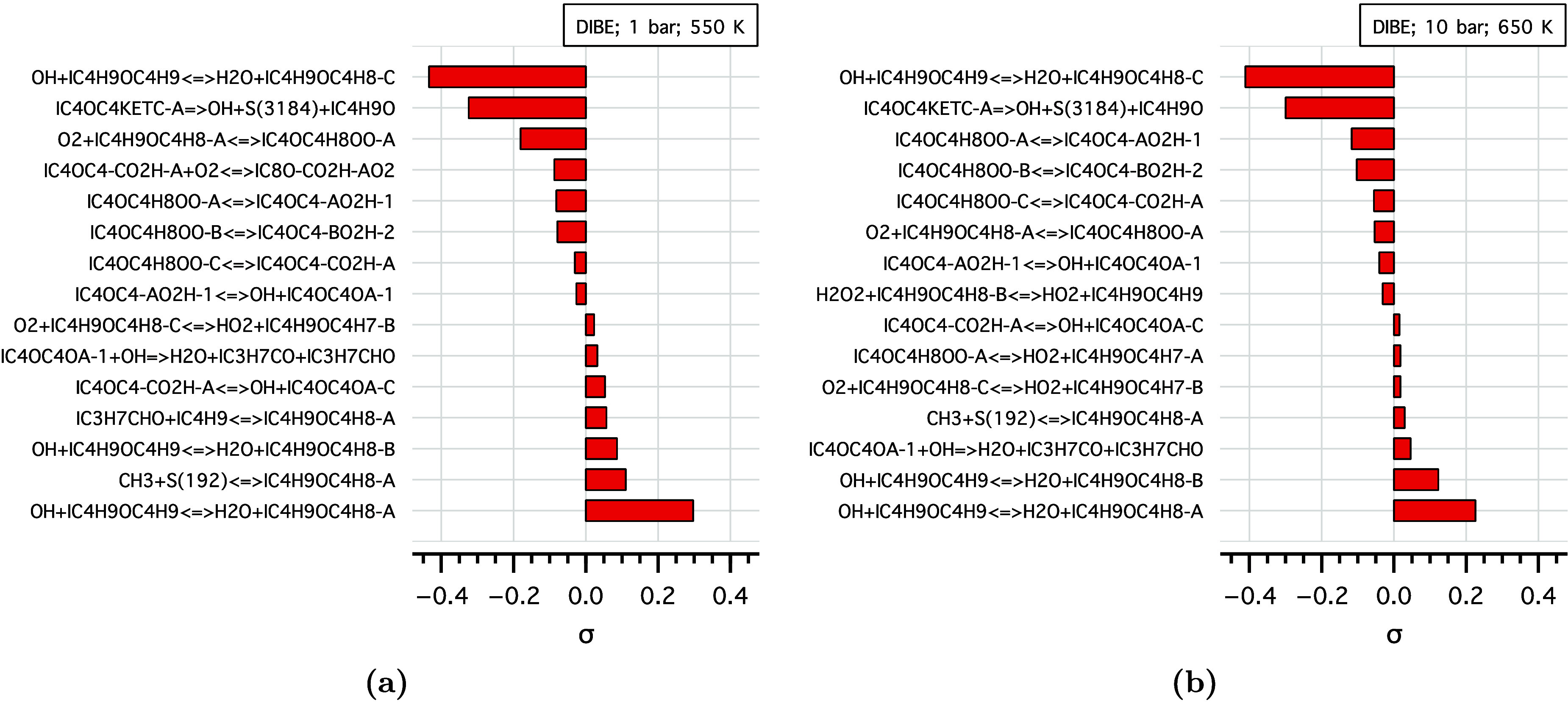
Species sensitivity analysis of DIBE at (a) 1 bar,550 K and (b) 10 bar, 650 K.

**Figure 8:**
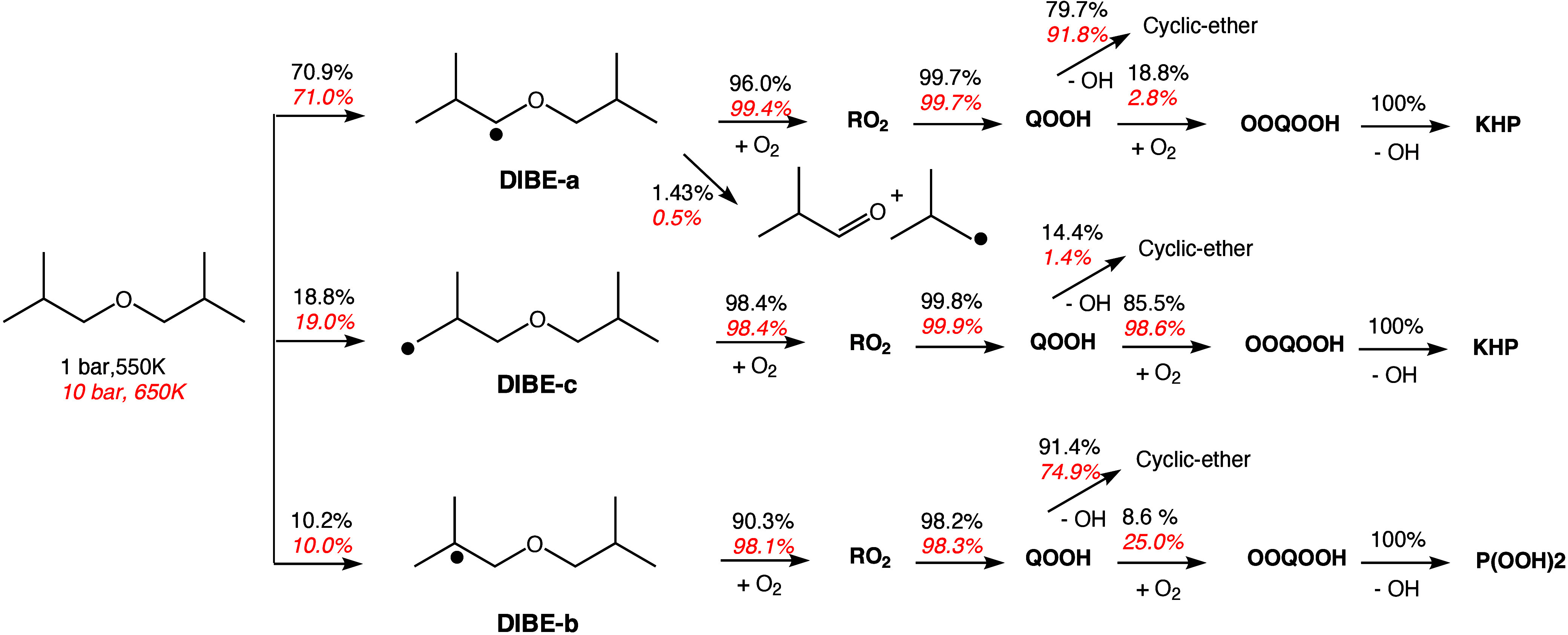
Flux analysis of DIBE at ϕ = 1 and 1 bar, 550 K (black) and 10 bar, 650 K (red, italic).

To understand the single NTC behavior of DIBE, sensitivity and flux analyses were carried out at 1 and 10 bar at different temperatures. The DIBE sensitivity and flux analysis results are shown in Figures 7 and 8, respectively, with the corresponding analysis pressures and temperatures. DIBE consumption is sensitive to H-abstraction reactions, particularly from the primary sites (DIBE-c, IC4H9OC4H8-C) and low-temperature chain branching pathways (first isomerization, second O_2_ addition, and KHP decomposition). The formation of cyclic ethers and olefins from this primary radical exhibits positive sensitivity, therefore contributing to the observed NTC behavior in the flow reactor. Interestingly, abstraction from the α-site to the ether’s functional group (to form IC4H9OC4H8-A radical) showed positive sensitivity, which can be attributed to the tendency of these radicals to form cyclic ether and undergo β-scission reactions at these conditions. Radical scission and intermediate decomposition reactions leading to isobutanal (IC3H7CHO) and its derivatives are among the sensitive reactions. The flux analysis in Figure 8, conducted at 10% consumption of DIBE, indicates that DIBE is consumed via H-abstraction reactions, with the α-radical to the ether functional group being the major radical formed (71%). This radical undergoes O_2_ addition and low-temperature chemistry leading to cyclic ether formation, which decomposes to isobutanal and its radical. At 20% DIBE consumption (not shown), approximately 40% of QOOH undergoes a second O_2_ addition, leading to KHP formation. This results in a low-temperature competition between chain-branching and chain-propagation reactions and thus contributes to the NTC behavior of DIBE. Similarly, the other two radicals that are formed undergo low-temperature chemistry and terminate via KHP or cyclic ether formation, leading to various branched intermediates such as isobutene, ketones, and isobutanol radicals. The tertiary radical (DIBE-b) is chemically constrained from forming KHP, and consequently, its low-temperature chemistry terminates via cyclic ether formation and alternative isomerization pathways at low and high pressure, respectively. The alternative isomerization pathway yields two OH radicals, unlike the cyclic ether formation, thereby contributing to the relatively higher reactivity observed at elevated pressure.

### 3.3 Di-*sec*-butyl Ether

**Figure 9:**
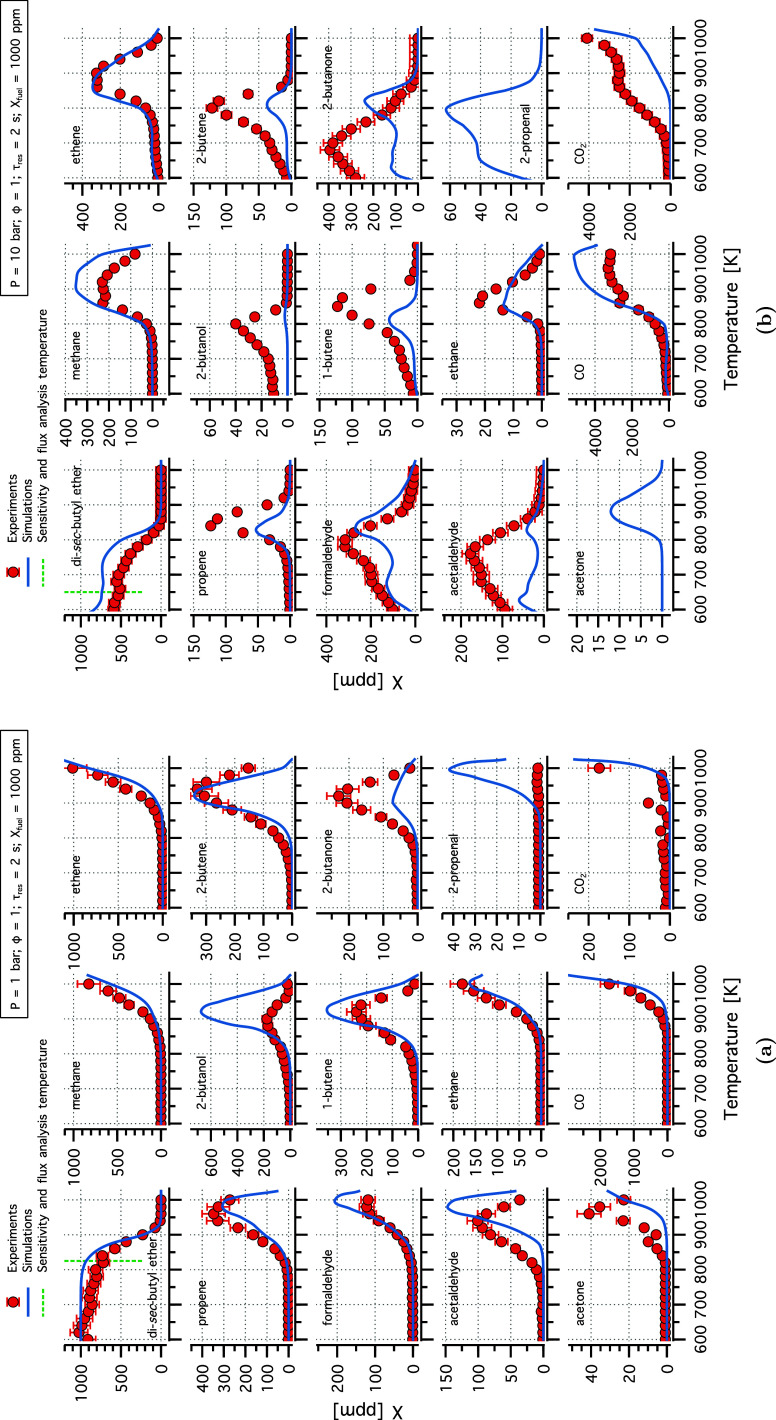
Experimental (symbols) and modeling (lines) mole fraction profiles of species identified in DSBE oxidation in flow reactor experiment at (a) 1 and (b) 10 bar, stoichiometric conditions, and a residence time of 2 s.

Figure 9 shows the experimental species profile compared to the chemical kinetic model predictions at 1 and 10 bar. Generally, the model agreed well with the experimental data, with no NTC behavior observed at 1 bar. The model underestimated 2-butanone and overestimated 2-propenal and *sec*-butanol (2-butanol). More discrepancies were observed at high pressure, where 1- and 2-butene, acetaldehyde, and 2-butanone were underestimated, and the peak is shifted approximately 100 K to higher temperatures for species, such as 2-butanone. Performing a sensitivity analysis at 750 K for acetaldehyde, 2-propenal, and 2-butanone revealed that alternative isomerization pathways (especially hydroperoxide cyclic ether formation) and the β-scission of the DSBE-d radical significantly impact the production of these intermediates. The use of rate constants in analogy to alkanes or shorter ethers may introduce inaccuracies, contributing to the observed discrepancy.

Similar to the results for DIBE, DSBE intermediates are formed at lower temperatures with higher production rates at higher pressure. We applied ROP analysis to investigate formaldehyde (CH_2_O), which peaks at 980 K (122 ppm) and 780 K (316 ppm) at 1 and 10 bar, respectively. In both conditions, formaldehyde is predominantly formed through subsequent reactions of methyl (CH_3_) radicals, with only about 8% directly produced from the dissociation of fuel intermediates at high pressure. Likewise, CH_3_ radicals can be produced through base chemistry reactions or from DSBE low-temperature intermediates, where the latter contributes to 12% and 73% of CH_3_ formed at 1 and 10 bar, respectively. This explains the production of formaldehyde at lower temperatures and with higher production rates at high pressure. At 1 bar, 90% of the fuel undergoes unimolecular decomposition to form butene and *sec*-butanol, where these two intermediates contribute only about 40% of the formed CH_3_ radicals. The remaining 60% CH_3_ is produced from 10% of the fuel, justifying the low production rates of CH_2_O at low pressures.

**Figure 10:**
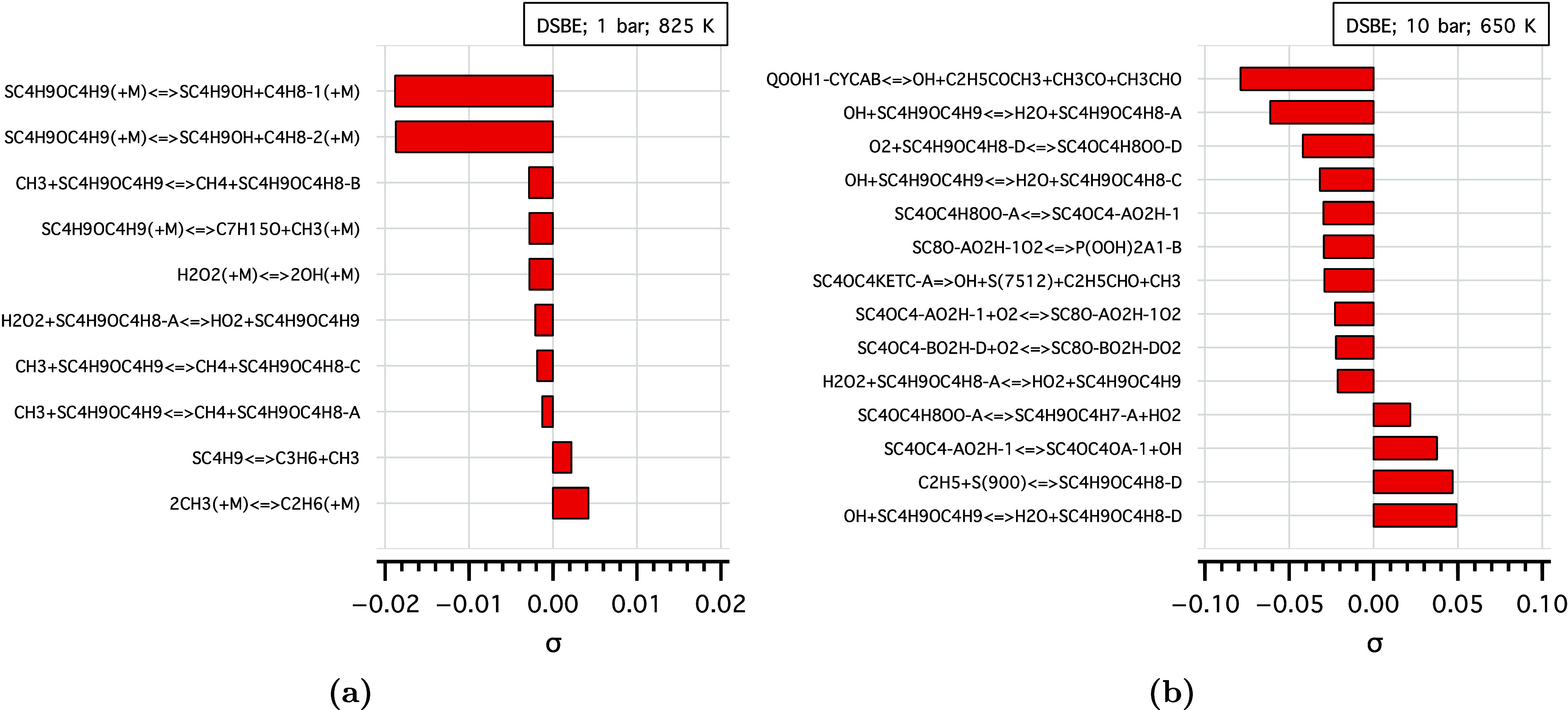
Species sensitivity analysis of DSBE at (a) 1 bar, 825 K and (b) 10 bar, 650 K.

**Figure 11:**
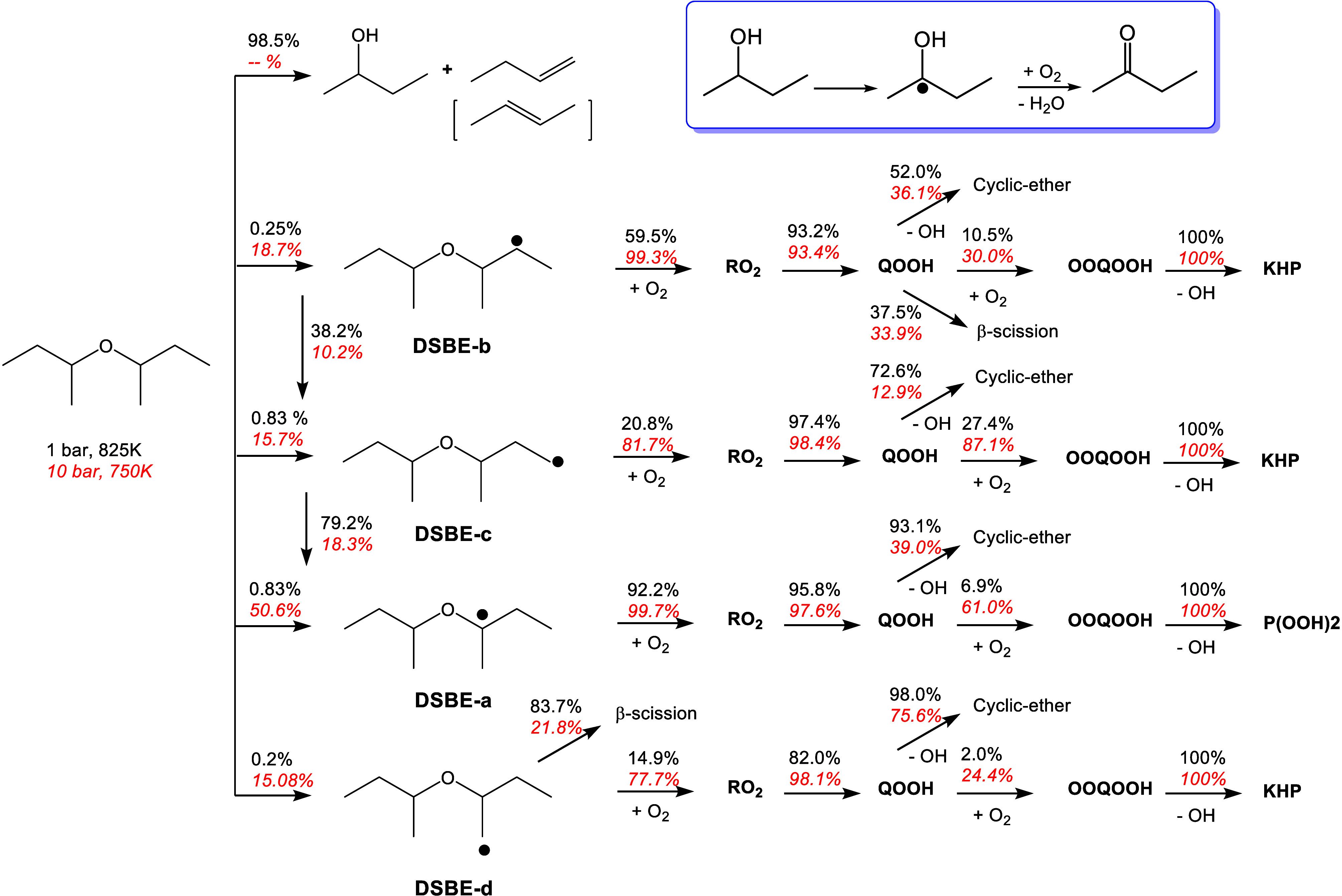
Flux analysis of DSBE 1 bar, 825 K (black) and 10 bar, 750 K (red, italic). The inset illustrates the major consumption pathway of *sec*-butanol.

Figure 10 shows the species sensitivity analysis of DSBE at 1 and 10 bar. The analysis indicates that at 1 bar and 825 K, DSBE consumption is exclusively sensitive to the unimolecular decomposition of DSBE to *sec*-butanol (SC4H9OH) and butene (1-butene, C4H8-1 or 2-butene, C4H8-2). The flux analysis in Figure 11 also shows that 98.5% of DSBE decomposes via those channels at 1 bar, which explains the absence of NTC behavior under these conditions. The major product *sec*-butanol, reaching 176 ppm at 900 K, contributed to the lower DSBE reactivity at low pressure and the absence of an NTC region. Flux analysis under the same conditions showed that the major *sec*-butanol radical (α to the OH functional group) undergoes chain-propagation reactions to form 2-butanone (C2H5COCH3) and water, competing with the low-temperature chemistry. This competition reduces reactivity and results in non-NTC behavior of DSBE. At high pressure, DSBE radicals undergo low-temperature chemistry with a major flux to the tertiary DSBE α-radical, which eventually undergoes alternative isomerization reactions to form P(OOH)_2_ radicals (P(OOH)2A1-B) and the subsequent hydroperoxide cyclic ethers (QOOH1-CYCAB), as can be seen in the sensitivity analysis at 10 bar (Figure 10(b)). The latter will decompose and form 2-butanone.

### 3.4 Summary Analysis

**Figure 12:**
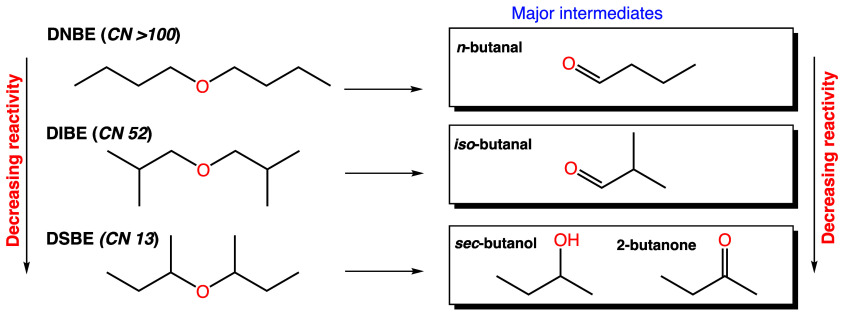
Key intermediates formed from the DBE isomers and their reactivity trend.

The chemical kinetic analyses indicated that the combustion behavior of the different ethers was influenced by key intermediate species that affected the available low-temperature reaction pathways. The structure of the parent molecules significantly affected the formed intermediate pool, as illustrated in Figure 12. Based on the experimentally detected species, sensitivity, and flux analysis, *n*-butanal and isobutanal are among the major intermediates formed from DNBE and DIBE at 1 bar, respectively. The position of the branch in DSBE, specifically α to the ether functional group, makes the unimolecular decomposition to form butene and *sec*-butanol influence its reactivity at low pressure. At high pressure, DSBE undergoes low-temperature chemistry, forming 2-butanone as one of the major intermediates. The key intermediates can provide insight into the observed reactivity trends of the DBE isomers. Alcohols and ketones are known to be less reactive than aldehydes,^42^ and the presence of branching in molecules tends to decrease reactivity,^43^ resulting in the reactivity trend of DSBE < DIBE < DNBE. Furthermore, it was demonstrated that the formation of *sec*-butanol strongly competes with the low-temperature chemistry of DSBE, resulting in the absence of NTC behavior at low pressure. The competition between the chain-propagation and chain-branching reactions of DNBE and DIBE results in the observed NTC behavior.

**Figure 13:**
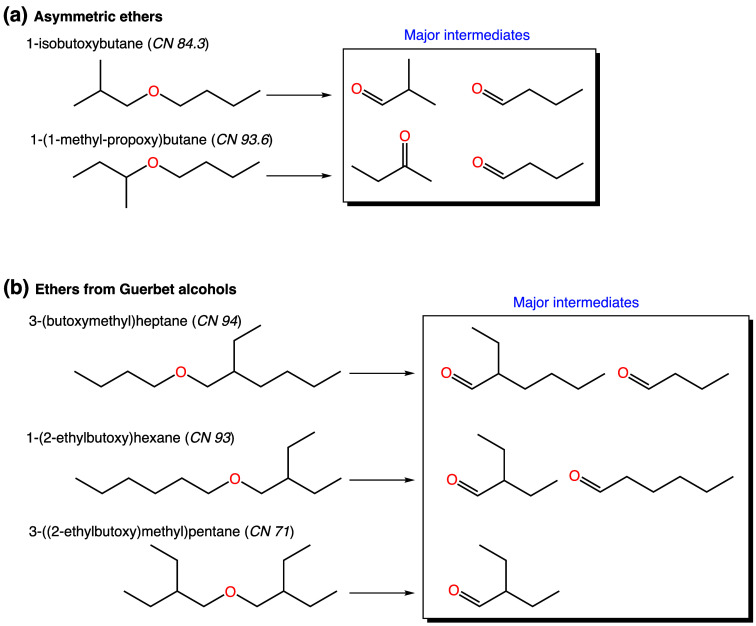
Key intermediates formed from (a) asymmetric alcohols and (b) ethers from Guerbet alcohols.

These findings can be extended to predict the reaction pathways of asymmetrical ethers and ethers made from Guerbet alcohols. The asymmetric nature in these ethers introduces additional complexity to their governing chemistry. 1-Isobutoxybutane and 1-(1-methyl-propoxy)butane ethers are structurally different because of the distinct branching position, β and α to the ether’s functional group, respectively. While we did not develop a kinetic model for these ethers, we predicted the major possible low-temperature pathways based on the predicted BDE,^39,40^ and considering 6-membered ring isomerizations only as illustrated in the flux analysis in the Supporting Information (Figure S9). Under these assumptions, the predominantly produced radicals from these asymmetric ethers are depicted in Figure 13a, aligning with experimental observations in the flow reactor experiments (Figures S7 and S8). However, other produced intermediates from the two asymmetric ethers are different, which affects the observed reactivity. Consequently, elucidating the reactivity trend of 1-isobutoxybutane and 1-(1-methyl-propoxy)butane, along with their measured ICNs of 84.3 and 93.6, respectively, is not readily evident.

On the other hand, for the Guerbet alcohol derived ethers, the branching position is consistently β to the ether’s functional group, as shown in Figure 13b. The key differences lie in the symmetry and chain length of the alkyl group on each side of the ether functional group. Predicting the main low-temperature intermediates for 3-(butoxymethyl)heptane and 1-(2-ethylbutoxy)hexane ethers, 2-ethylhexanal and 2-ethylbutanal emerge as the major aldehydes formed, respectively. The branching positions of 2-ethylhexanal and 2-ethylbutanal initiate similar chemistry, while the chain length affects the reactivity. Nevertheless, the overall parent ethers’ reactivity is balanced by the accompanying *n*-aldehyde intermediates, ensuring an equal number of reactive sites in both ethers and resulting in comparable ICNs of 93 and 94. For the 3-((2-ethylbutoxy)methyl)pentane ether in Figure 13b, the symmetrical nature of the molecule leads to 2-ethylbutanal as the major intermediate, resulting in relatively lower reactivity (ICN of 70) compared to that of the other ethers with the same number of carbon atoms .

The analysis above indicates that branching significantly influences the ether reactivity, similar to alkane chemistry. However, the branching position relative to the ether functional group has an even greater effect, as observed in the case of DBE and the asymmetrical ethers considered in this work.

## 4 Concluding Remarks

The combustion behavior of three isomers of DBE was studied experimentally in a flow reactor at 1 and 10 bar at a stoichiometric equivalence ratio to elucidate the structural effects on autoignition chemistry. Double, single, and no NTC behavior was observed for DNBE, DIBE, and DSBE, respectively, at atmospheric pressure. A DNBE model from the literature and newly developed DIBE and DSBE models showed good agreement with the experimental data in most cases. Flux and sensitivity analyses of the DBE isomers were carried out to elucidate their oxidation pathways. The analysis indicated that the governing chemistries that determined their reactivities and combustion behavior were influenced by their molecular structures and the key formed intermediates.

DNBE mostly formed an α-radical that decomposed via chain branching, chain propagation, or the scission of the QOOH radical pathways. The competing effects of these pathways results in the NTC behavior of DNBE. Similar to DNBE, the competition between the cyclic ether and the KHP formation in DIBE resulted in NTC behavior. For DSBE at low pressure, the unimolecular decomposition forming *sec*-butanol and 1-butene (or 2-butene) is the most sensitive and major pathway, causing the lowest reactivity among the DBE isomers and no NTC behavior.

These findings for the symmetric DBE isomers were then extended to asymmetric ethers and branched ethers made from Guerbet alcohols to obtain insights into their combustion behavior. This approach indicates that the ether reactivity is significantly influenced by branching, similar to alkane chemistry. However, more influential is the branching position relative to the ether’s functional group.

## Supporting Information Available

Uncertainty values, detailed schematics of the flow reactor, BDE comparisons, detailed flux analysis of ethers derived from Guerbet alcohols, and mole fraction profiles (PDF)
Speciation data, flow rates, and temperature profiles of the flow reactor, DIBE species dictionary, and DSBE species dictionary (XLSX)
DIBE chemical kinetic model (TXT)
DSBE chemicl kinetic model (TXT)
DIBE species dictionary (PDF)
DSBE species dictionary (PDF)
Arrhenius parameters for DIBE reactions (PDF)
