# Supplementary material for: Chemical Kinetics Investigations of Dibutyl Ether Isomers Oxidation in a Laminar Flow Reactor
Source: Energy Fuels. 2024 Oct 31;38(22):22501–15. doi: 10.1021/acs.energyfuels.4c03432 (PMC11586913; doi:10.1021/acs.energyfuels.4c03432)
Supplement: Supplementary file 1 — ef4c03432_si_001.pdf [file ef4c03432_si_001.pdf]

Supporting Information: Chemical kinetics investigations of dibutyl ether isomers oxidation in a laminar flow reactor

Nimal Naser,[†] Samah Y. Mohamed,[†] Gina M. Fioroni,[†] Seonah Kim,[†,‡] and Robert L. McCormick[*,†]

†*National Renewable Energy Laboratory, Golden, CO 80401, USA*

‡*Chemistry Department, Colorado State University, Fort Collins, CO 80523, USA*

* E-mail: robert.mccormick@nrel.gov

Table S1: Uncertainty values used in uncertainty quantification.

Parameter/Property	Value
Reactor length [m]	$\pm$ 0.020
Reactor diameter [m]	$\pm$ 0.0020
Pressure [bar]	$\pm$ 0.1
Temperature uncertainty [K]	$\pm$ 6
Fuel delivery uncertainty [μL/min]	1.0
Diluent flow uncertainty [SLPM]	0.1
Oxidizer flow uncertainty [SCCM]	1.0
Diluent density [kg/m^3]	0.01
Oxidizer density [kg/m^3]	0.01

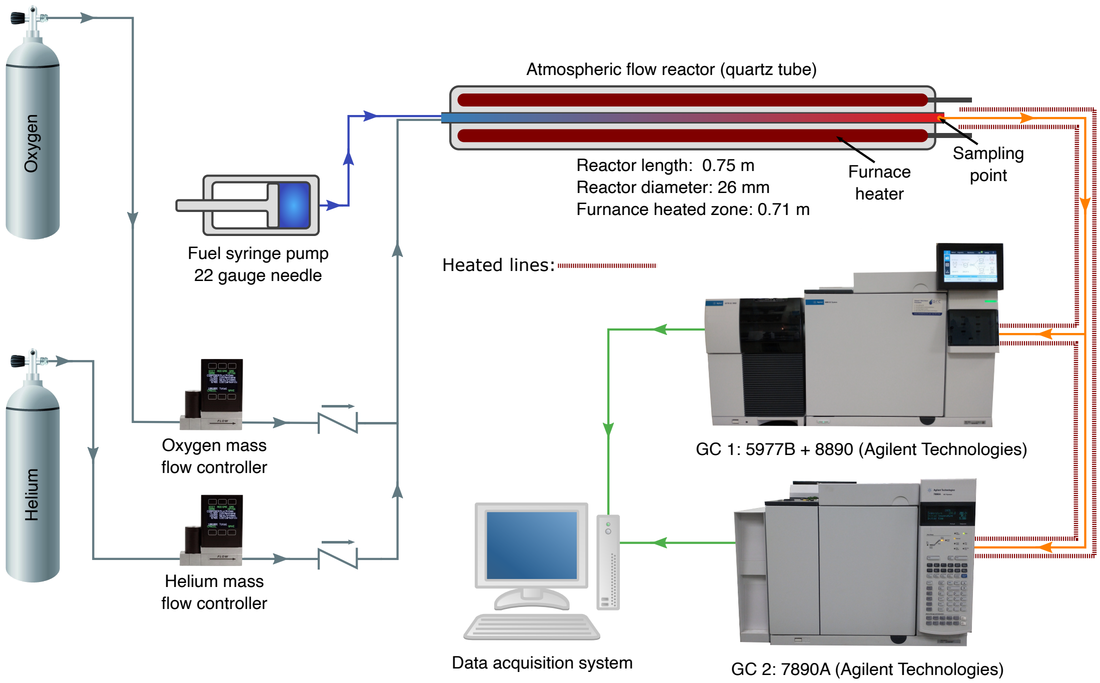

Figure S1: Schematic of the atmospheric pressure flow reactor at NREL.

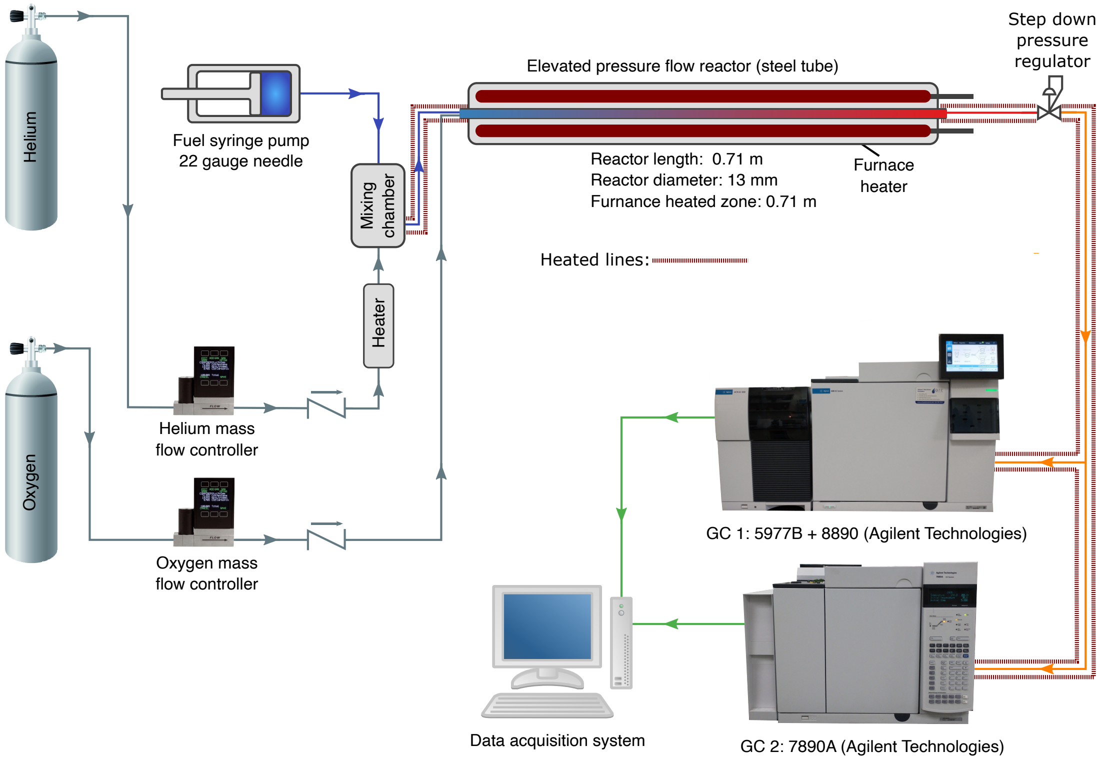

Figure S2: Schematic of the elevated pressure flow reactor at NREL.

Speciation data

Available as spreadsheet.

Temperature profile of NREL flow reactor

Available as spreadsheet.

BDEs comparisons

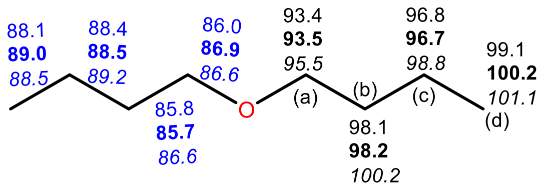

(a)

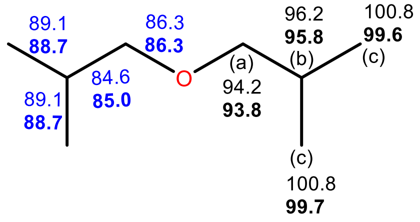

(b)

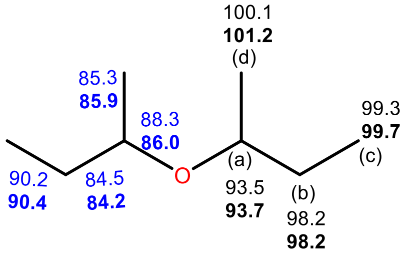

(c)

Figure S3: Calculated (normal font), predicted (bold) and calculated (italic) BDE of DNBE (top left), DIBE (top right), and DSBE (bottom). Values in blue and black indicate BDE in kcal/mol for C-C (or C-O) and C-H bonds, respectively.

The calculated BDEs in this study are compared to predictions from ALFABET BDE prediction model,[39,40] and G3B3 calculations for DNBE from the literature.[17] ALFABET prediction tool[39,40] is based on automated denisty functional theory (DFT) calculations at the M06-2X/def2-TZVP level of theory for 42,577 small organic molecules, resulting in 290,664 BDEs. It has a mean error of 0.58 kcal/mole for molecules not in the training set. These predictions were compared with the calculations from this study, and differences were primarily in the range of 0.0–1.2 kcal/mol. However, notable difference of 2.3 kcal/mol was observed for C-O BDE of DSBE. Our calculations were also compared to the calculated BDE of DNBE at G3B3 from Thion et al.[17] (italic values). For both C-C or C-O bonds, calculated BDEs were within 2.1 kcal/mol from Thion et al.[17] calculated values which can be attributed to the different used level of theories.

Detailed flux analysis of DBE isomers

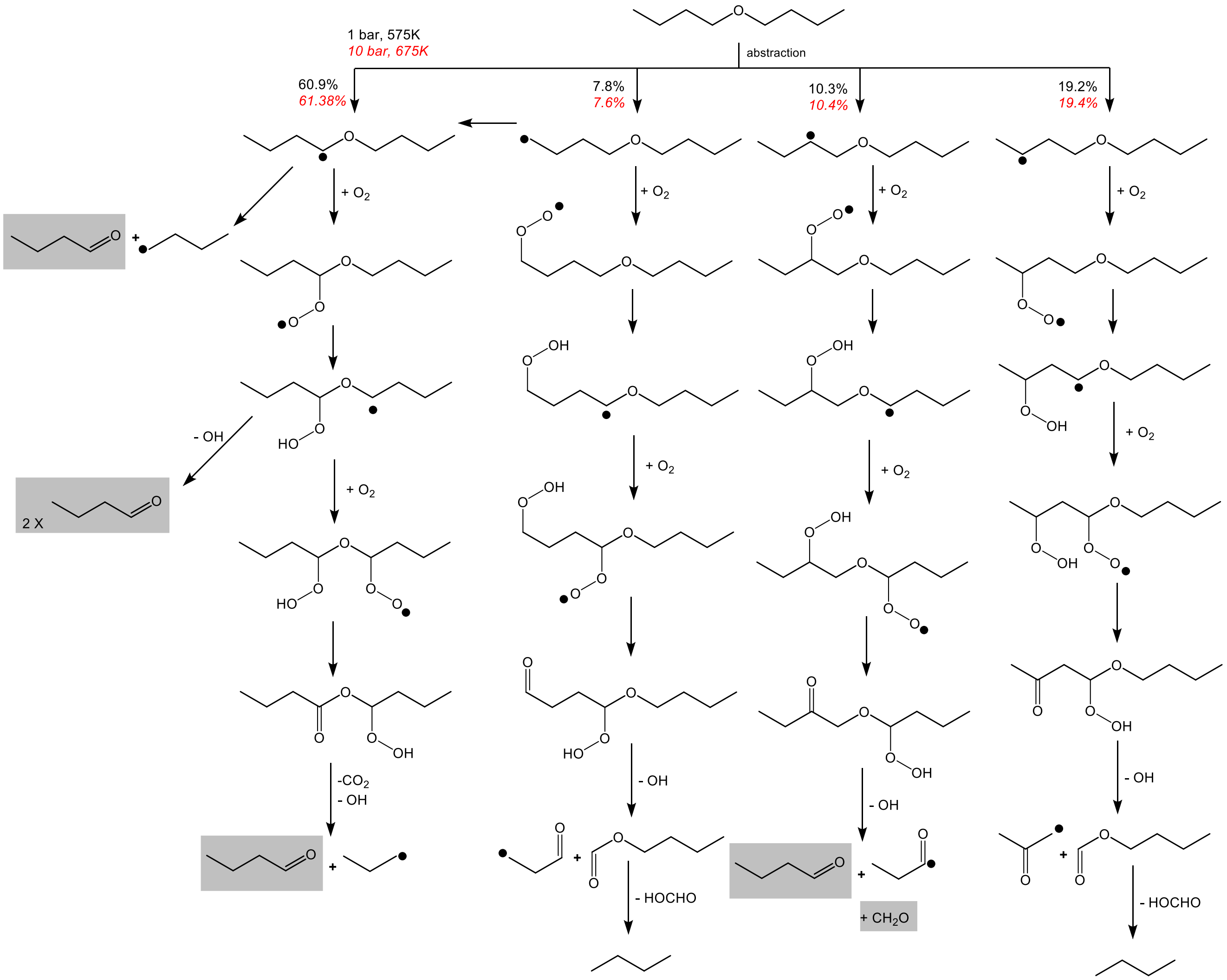

Figure S4: Flux analysis of DNBE at $\phi = 1$, 1 bar, 575 K (black) and 10 bar, 675 K (red, italic).

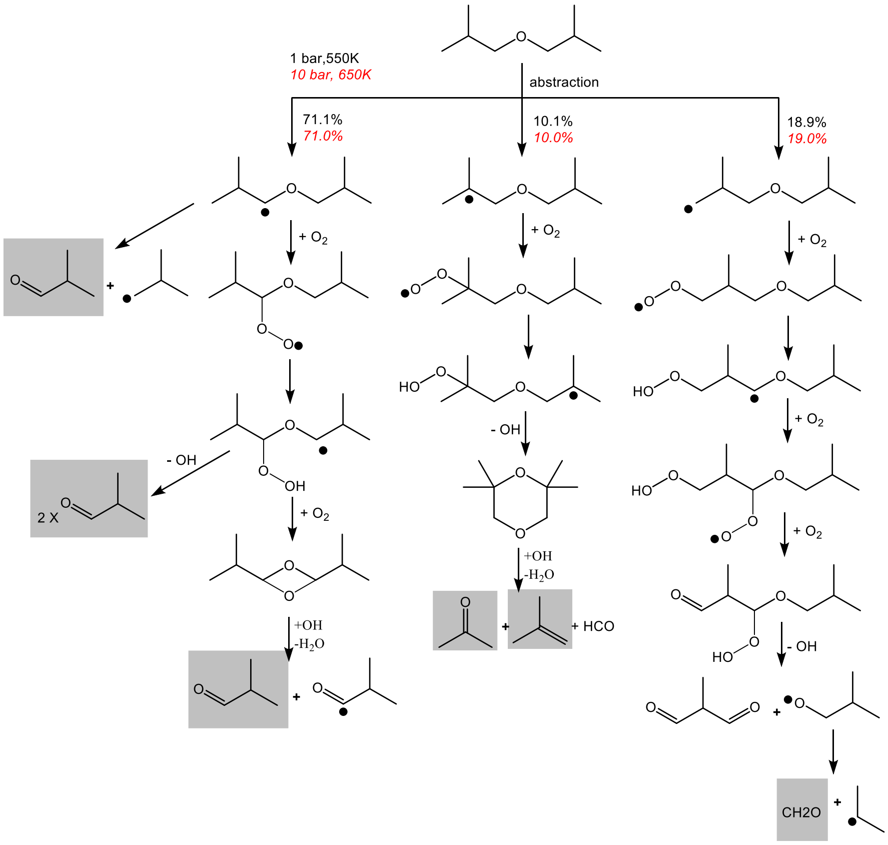

Figure S5: Flux analysis of DIBE at $\phi = 1$, 1 bar, 550 K (black) and 10 bar, 650 K (red, italic).

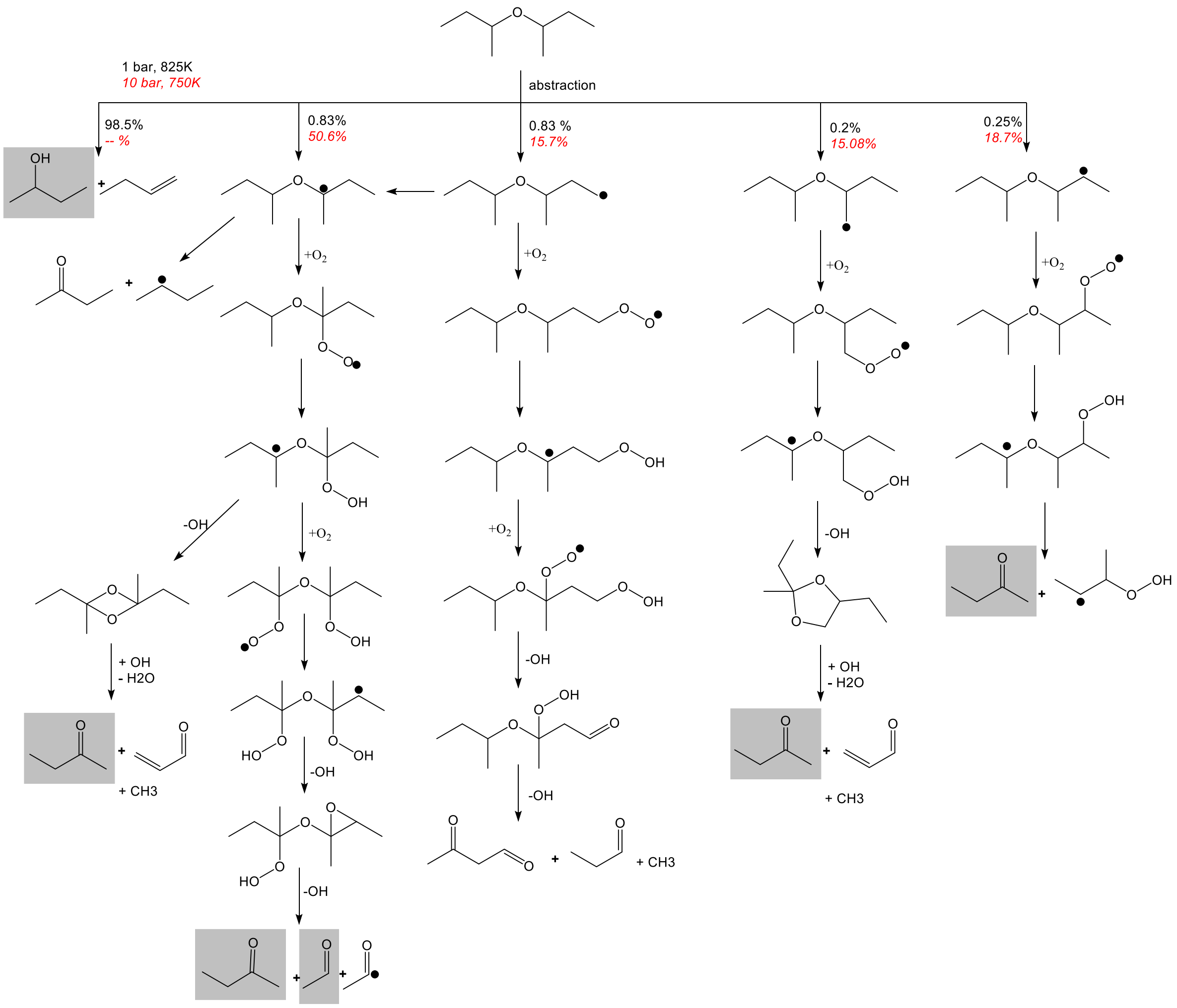

Figure S6: Flux analysis of DSBE at $\phi = 1$, 1 bar, 825 K (black) and 10 bar, 750 K (red, italic).

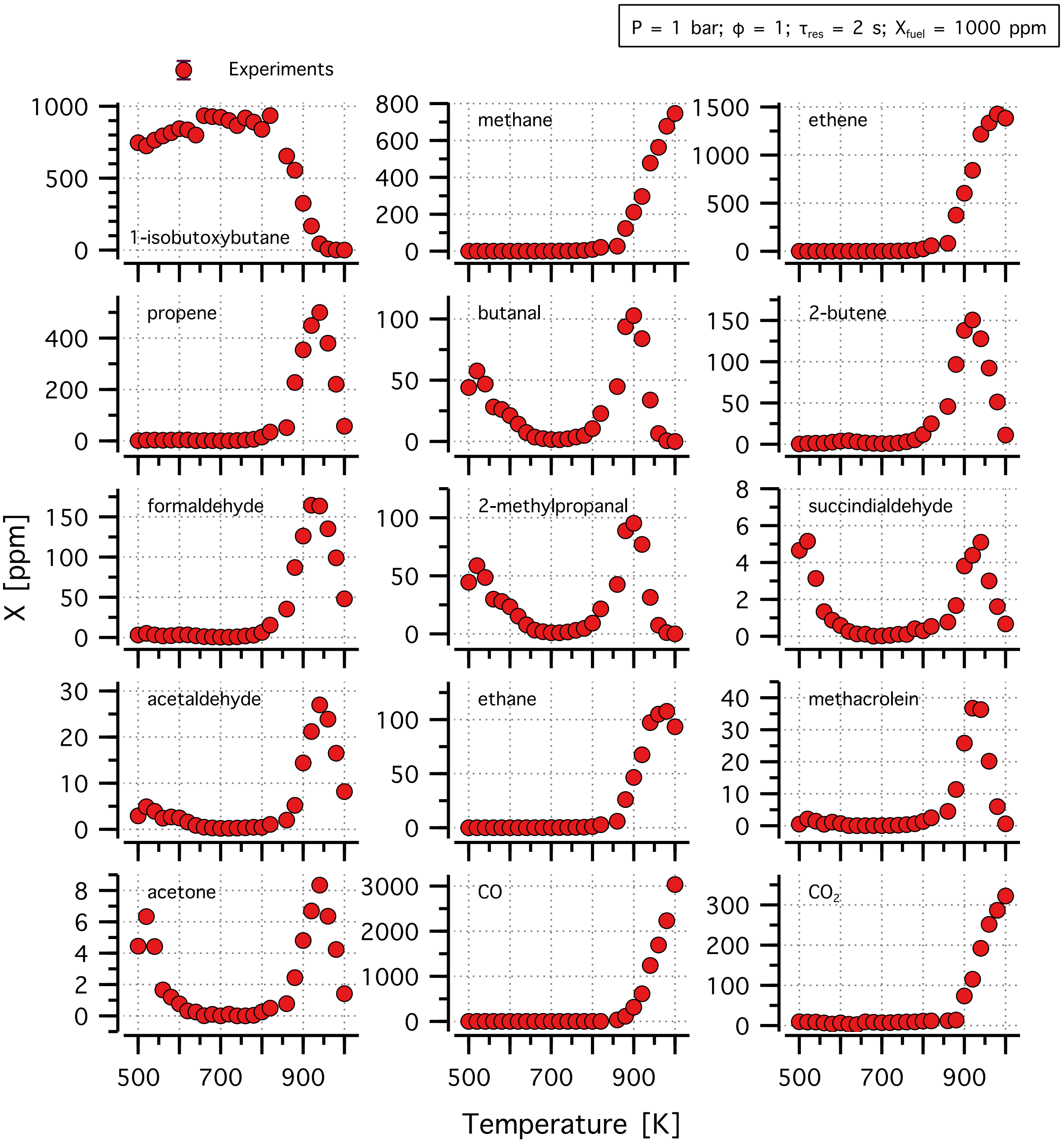

Figure S7: Experimental mole fraction profiles of species identified in 1-isobutoxybutane oxidation in flow reactor experiment at 1 bar, stoichiometric conditions, and a residence time of 2 s.

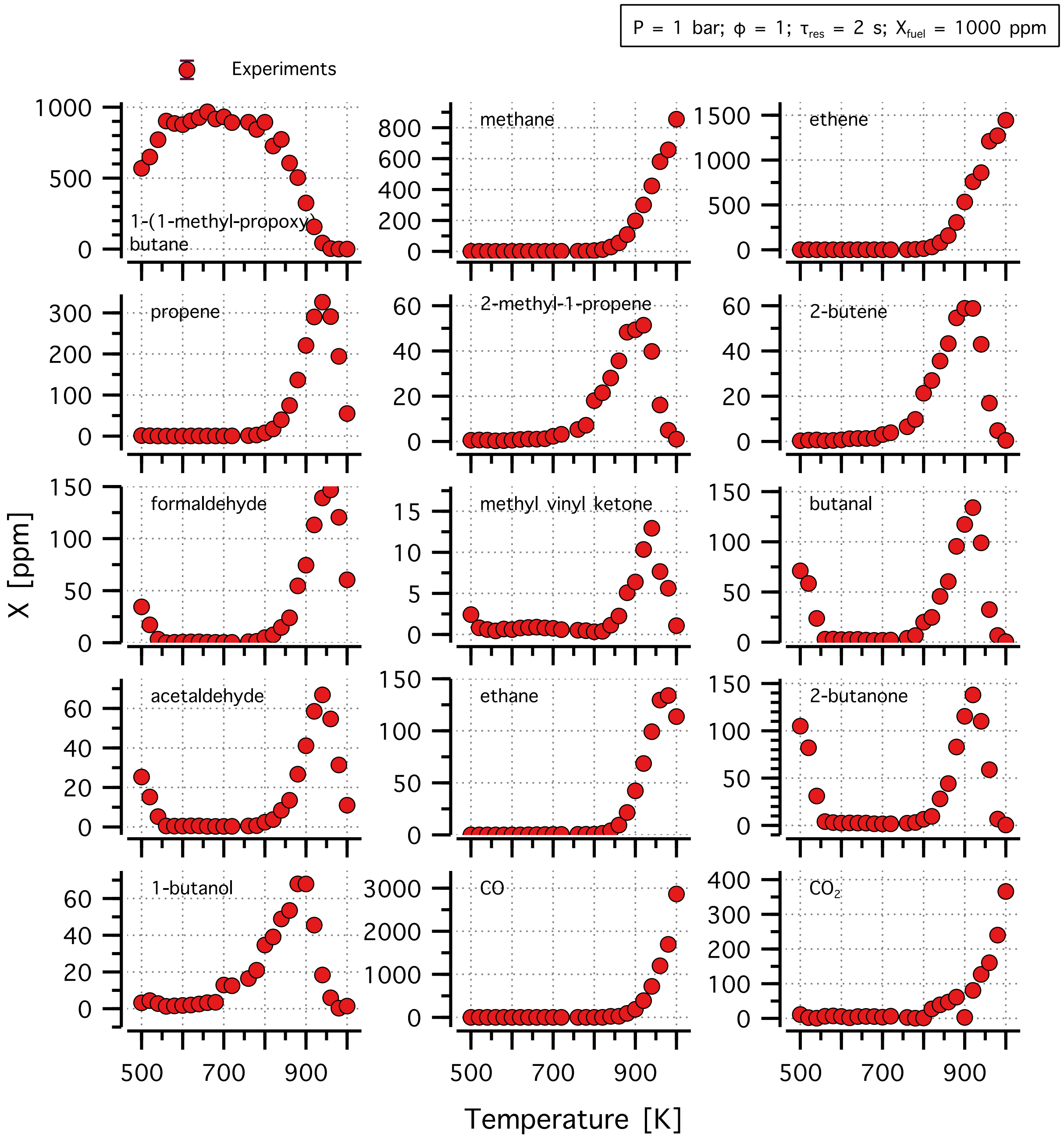

Figure S8: Experimental mole fraction profiles of species identified in 1-(1-methyl-propoxy)butane oxidation in flow reactor experiment at 1 bar, stoichiometric conditions, and a residence time of 2 s.

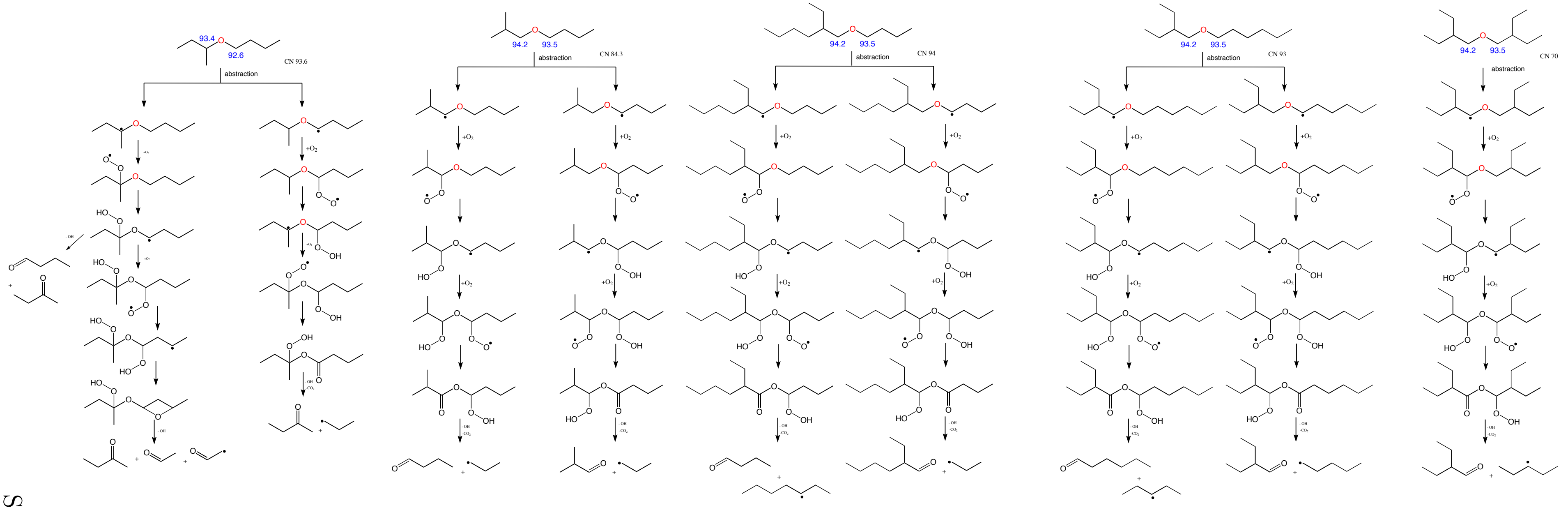

Figure S9: Flux analysis of asymmetric and ethers derived from Guerbet alcohols.
