# Supplementary material for: Chemical Kinetics Investigations of Dibutyl Ether Isomers Oxidation in a Laminar Flow Reactor
Source: Energy Fuels. 2024 Oct 31;38(22):22501–15. doi: 10.1021/acs.energyfuels.4c03432 (PMC11586913; doi:10.1021/acs.energyfuels.4c03432)
Supplement: Supplementary file 5 — ef4c03432_si_005.pdf [file ef4c03432_si_005.pdf]

Chemical kinetics investigations of dibutyl ether isomers oxidation in a laminar flow reactor

Nimal Naser,† Samah Y. Mohamed, † Gina M. Fioroni, † Seonah Kim,†,‡ and Robert L. McCormick*,†

† *National Renewable Energy Laboratory, Golden, CO 80401, USA*
‡ *Chemistry Department, Colorado State University, Fort Collins, CO 80523, USA*

* E-mail: robert.mccormick@nrel.gov

di-iso-butyl ether **(DIBE) species dictionary**

**!Fuel and radicals**		
IC4H9OC4H9	CC(C)COCC(C)C	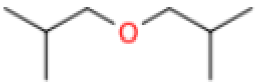
IC4H9OC4H8-A	CC(C)[CH]OCC(C)C	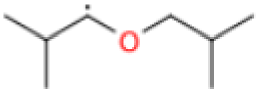
IC4H9OC4H8-B	C[C](C)COCC(C)C	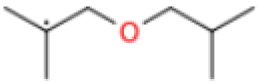
IC4H9OC4H8-C	[CH2]C(C)COCC(C)C	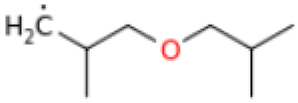
!Olefins and radicals		
IC4H9OC4H7-A	C/C(C)=C/OCC(C)C	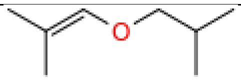
IC4H9OC4H7-B	CC(COCC(C)C)=C	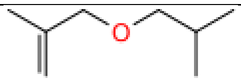
**!RO2**		
IC4OC4H8OO-A	CC(C)C(O[O])OCC(C)C	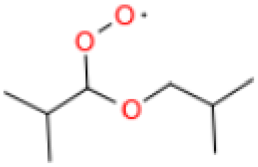
IC4OC4H8OO-B	CC(C)(O[O])COCC(C)C	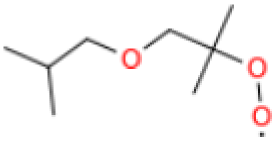

IC4OC4H8OO-C	CC(COCC(C)C)CO[O]	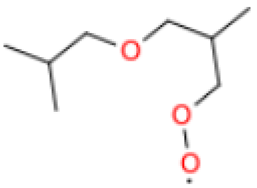
**!QOOH**		
IC4OC4-AO2H-B	C[C](C)C(OO)OCC(C)C	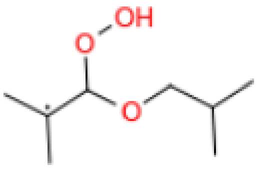
IC4OC4-AO2H-C	CC([CH2])C(OO)OCC(C)C	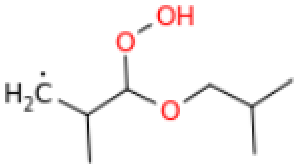
IC4OC4-AO2H-1	CC(C)C(OO)O[CH]C(C)C	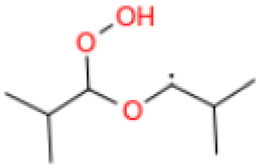
IC4OC4-AO2H-2	CC(C)C(OO)OC[C](C)C	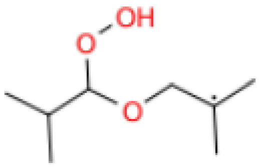
IC4OC4-AO2H-3	CC(C)C(OO)OCC(C)[CH2]	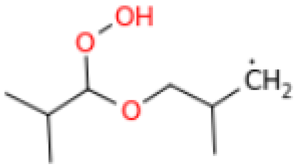
IC4OC4-BO2H-A	CC(C)(OO)[CH]OCC(C)C	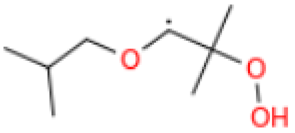
IC4OC4-BO2H-1	CC(C)(OO)CO[CH]C(C)C	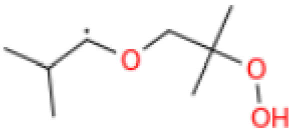
IC4OC4-BO2H-2	CC(C)(OO)COC[C](C)C	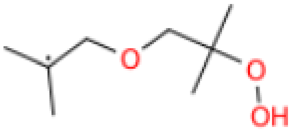

IC4OC4-CO2H-A	CC([CH]OCC(C)C)COO	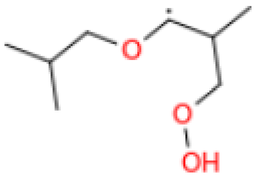
IC4OC4-CO2H-B	C[C](COCC(C)C)COO	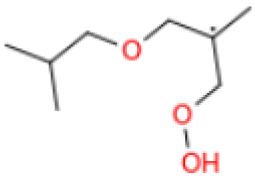
IC4OC4-CO2H-C	[CH2]C(COCC(C)C)COO	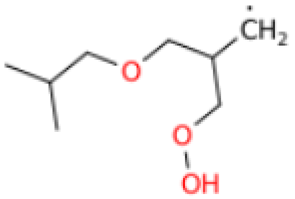
**!Cyclic-ethers**		
IC4OC4OA-B	CC1(C(O1)OCC(C)C)C	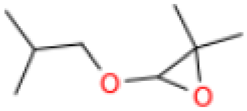
IC4OC4OA-C	CC1COC1OCC(C)C	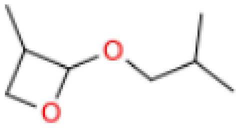
IC4OC4OA-1	CC(C)C(O1)OC1C(C)C	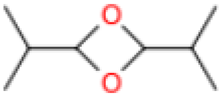
IC4OC4OA-2	CC([C@H](COC1)OCC1(C)C)C	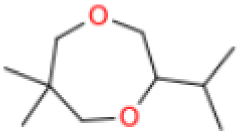
IC4OC4OA-3	CC([C@H]1OC[C@H](CCOC1)C)C	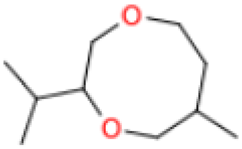
IC4OC4OB-C	CC1(COCC(C)C)CO1	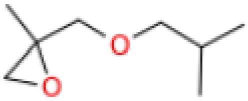
IC4OC4OB-1	CC(CO[C@@H]1C(C)C)(COC1)C	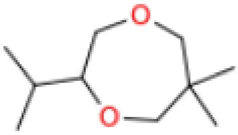

IC4OC4OB-2	CC1(C)COCC(C)(C)O1	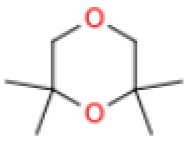
IC4OC4OC-C	CC(C)COCC1COC1	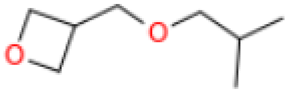
**!QOOH**		
IC8O-AO2H-BO2	CC(C(OO)OCC(C)C)(O[O])C	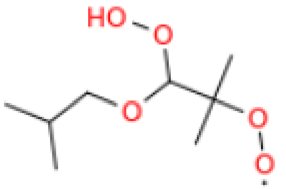
IC8O-AO2H-CO2	CC(C(OO)OCC(C)C)CO[O]	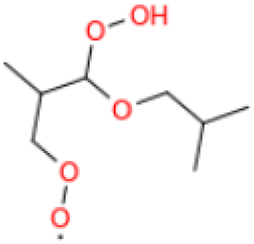
IC8O-AO2H-1O2	CC(C(OO)OC(O[O])C(C)C)C	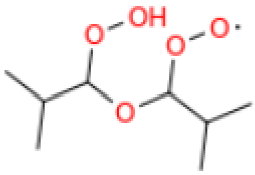
IC8O-AO2H-2O2	CC(C(OO)OCC(C)(O[O])C)C	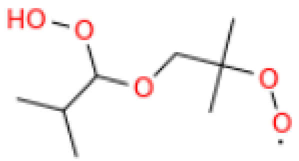
IC8O-AO2H-3O2	CC(C(OO)OCC(CO[O])C)C	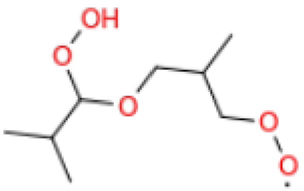
IC8O-BO2H-AO2	CC(C(O[O])OCC(C)C)(OO)C	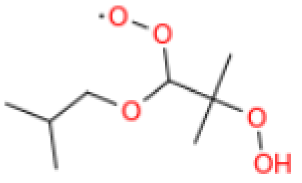

IC8O-BO2H-1O2	CC(COC(O[O])C(C)C)(OO)C	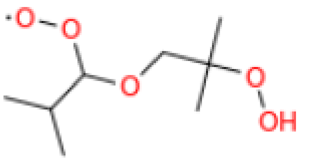
IC8O-BO2H-2O2	CC(COCC(C)(O[O])C)(OO)C	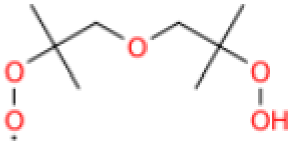
IC8O-CO2H-AO2	CC(C(O[O])OCC(C)C)COO	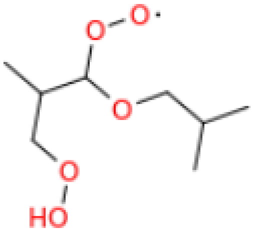
IC8O-CO2H-BO2	CC(COCC(C)C)(O[O])COO	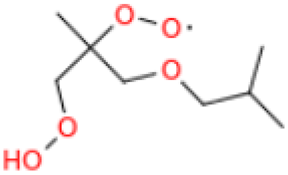
IC8O-CO2H-CO2	CC(C)COCC(CO[O])COO	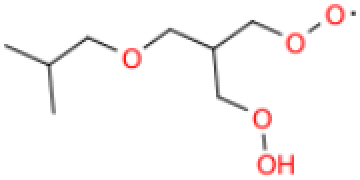
**!KHP**		
IC4OC4KETA-B	CC(OO)(C)C(OCC(C)C)=O	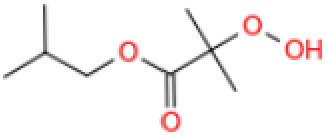
IC4OC4KETA-C	O=C(OCC(C)C)C(C)COO	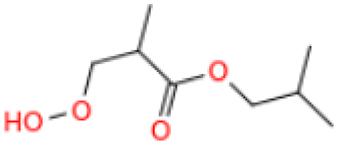
IC4OC4KETA-1	CC(C)C(OC(OO)C(C)C)=O	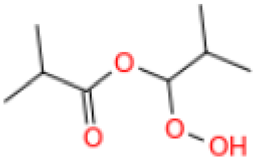

IC4OC4KETA-2	CC(C)C(OCC(C)(OO)C)=O	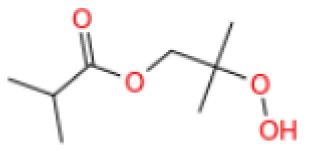
IC4OC4KETA-3	CC(C)C(OCC(C)COO)=O	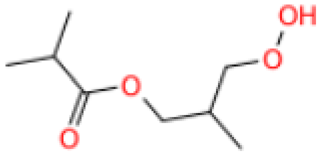
IC4OC4KETC-A	CC(C)COC(OO)C(C)C=O	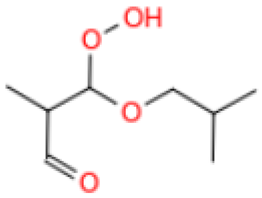
IC4OC4KETC-B	CC(C)COC[C@@](C)(OO)C=O	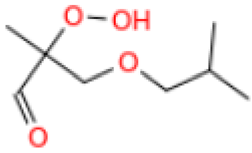
IC4OC4KETC-C	CC(C)COCC(COO)C=O	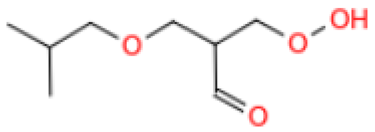
**!P(OOH)2 and its cyclic ether**		
P(OOH)2BA-1	CC(C)(OO)C(OO)O[CH]C(C)C	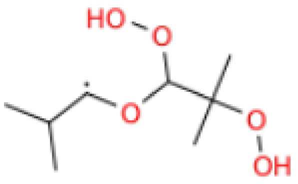
P(OOH)2B1-A	CC(C)(OO)[CH]OC(OO)C(C)C	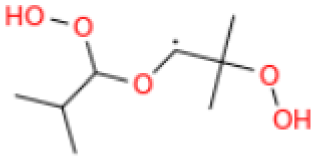
P(OOH)2B2-A	CC(C)(OO)[CH]OCC(C)(OO)C	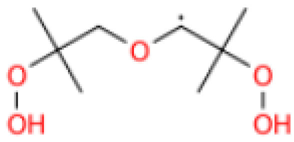
QOOHB-CYCA1	CC(C)C1OC(O1)C(C)(OO)C	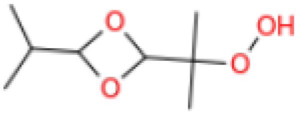

QOOHB-CYCA2	CC1(C)CO[C@@H](COC1)C(C)(OO)C	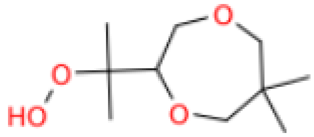
**!intermediates**		
iBuOH(183)	CC(C)CO	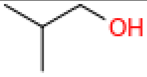
S(177)	[CH2]OCC(C)C	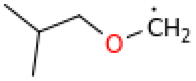
S(178)	C[CH]COCC(C)C	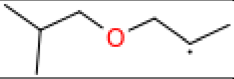
S(192)	CC=COCC(C)C	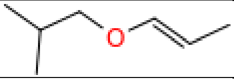
S(224)	C=CCOCC(C)C	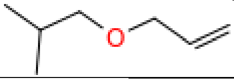
S(3184)	CC(C=O)C=O	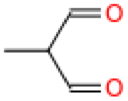
