# Supplementary material for: Chemical Kinetics Investigations of Dibutyl Ether Isomers Oxidation in a Laminar Flow Reactor
Source: Energy Fuels. 2024 Oct 31;38(22):22501–15. doi: 10.1021/acs.energyfuels.4c03432 (PMC11586913; doi:10.1021/acs.energyfuels.4c03432)
Supplement: Supplementary file 6 — ef4c03432_si_006.pdf [file ef4c03432_si_006.pdf]

Chemical kinetics investigations of dibutyl ether isomers oxidation in a laminar flow reactor

Nimal Naser,† Samah Y. Mohamed, † Gina M. Fioroni, † Seonah Kim,†,‡ and Robert L. McCormick*,†

† *National Renewable Energy Laboratory, Golden, CO 80401, USA*
‡ *Chemistry Department, Colorado State University, Fort Collins, CO 80523, USA*

* E-mail: robert.mccormick@nrel.gov

di-sec-butyl ether **(DSBE) species dictionary**

**!Fuel and radicals**		
SC4H9OC4H9	CCC(C)OC(C)CC	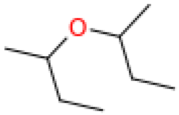
SC4H9OC4H8-A	CCC(C)O[C](C)CC	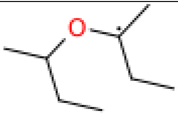
SC4H9OC4H8-B	CCC(C)OC(C)[CH]C	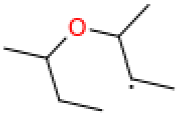
SC4H9OC4H8-C	CCC(C)OC(C)C[CH2]	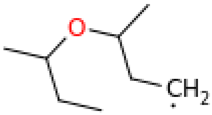
SC4H9OC4H8-D	CCC([CH2])OC(C)CC	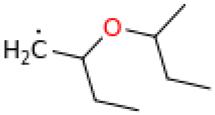
**!Olefins and radicals**		
SC4H9OC4H7-A	CCC(C)O/C(C)=C/C	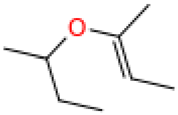
SC4H9OC4H7-B	CCC(C)OC(C)C=C	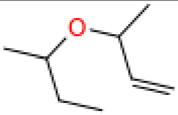
SC4H9OC4H7-D	CCC(C)OC(CC)=C	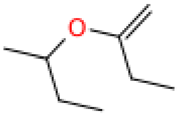
**!RO2**		

SC4OC4H8OO-A	CCC(C)OC(O[O])(C)CC	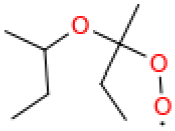
SC4OC4H8OO-B	CCC(C)OC(C)C(O[O])C	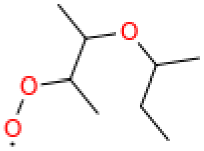
SC4OC4H8OO-C	CCC(C)OC(C)CCO[O]	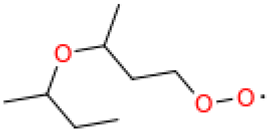
SC4OC4H8OO-D	CCC(C)OC(CO[O])CC	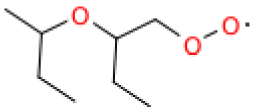
**!QOOH**		
SC4OC4-AO2H-1	CC[C](C)OC(OO)(C)CC	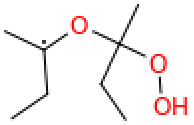
SC4OC4-AO2H-2	C[CH]C(C)OC(OO)(C)CC	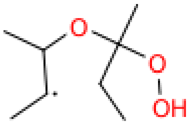
SC4OC4-AO2H-C	CCC(C)OC(OO)(C)C[CH2]	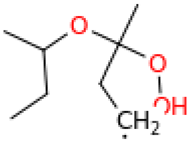
SC4OC4-BO2H-A	CCC(C)O[C](C)C(OO)C	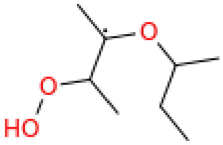
SC4OC4-BO2H-D	CCC(C)OC([CH2])C(OO)C	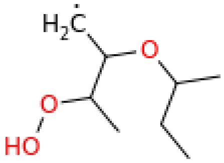
SC4OC4-BO2H-1	CC[C](C)OC(C)C(OO)C	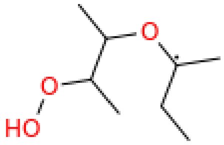

SC4OC4-DO2H-B	CCC(C)OC(COO)[CH]C	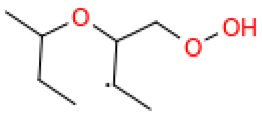
SC4OC4-DO2H-C	CCC(C)OC(COO)C[CH2]	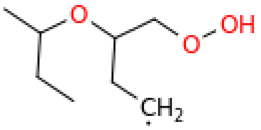
SC4OC4-DO2H-1	CC[C](C)OC(COO)CC	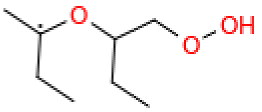
SC4OC4-CO2H-A	CCC(C)O[C](C)CCOO	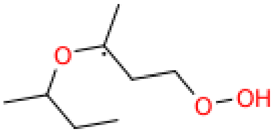
SC4OC4-CO2H-D	CCC(C)OC([CH2])CCOO	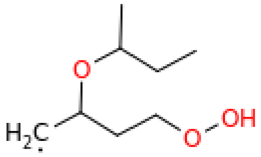
**!Cyclic-ethers**		
SC4OC4OA-C	CCC(C)OC1(C)CCO1	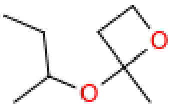
SC4OC4OA-1	CC[C@](C)(O1)O[C@@]1(C)CC	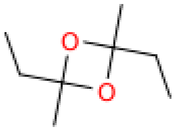
SC4OC4OA-2	C[C@H](CO1)[C@@H](C)O[C@]1(C)CC	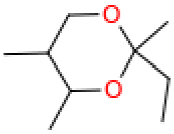
SC4OC4OB-A	CCC(C)OC(C)(O1)C1C	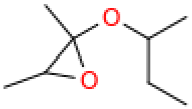
SC4OC4OB-D	CCC(C)OC(CO1)C1C	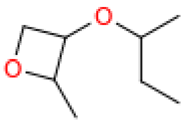
SC4OC4OC-D	CCC(C)OC1CCOC1	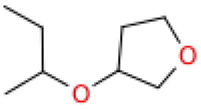

SC4OC4OD-1	CCC1(C)OC(CO1)CC	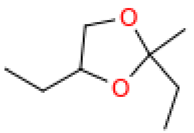
**!O2QOOH**		
SC8O-AO2H-CO2	CCC(C)O[C@@](C)(OO)CCO[O]	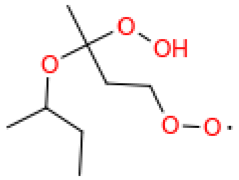
SC8O-AO2H-1O2	CC[C@@](C)(O[O])O[C@@](C)(OO)CC	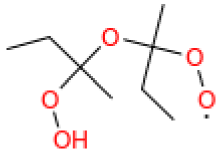
SC8O-AO2H-2O2	CC(O[O])C(C)O[C@@](C)(OO)CC	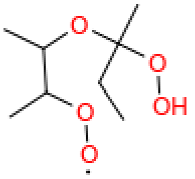
SC8O-BO2H-AO2	CC(OO)[C@@](C)(O[O])OC(C)CC	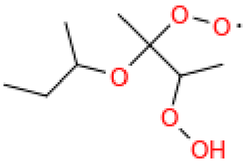
SC8O-BO2H-DO2	CC(OO)C(CO[O])OC(C)CC	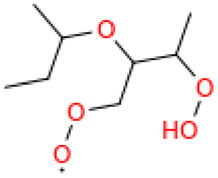
SC8O-BO2H-1O2	CC(OO)C(C)O[C@@](C)(O[O])CC	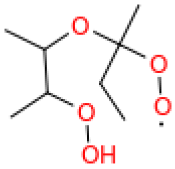
SC8O-DO2H-BO2	CCC(C)OC(COO)C(O[O])C	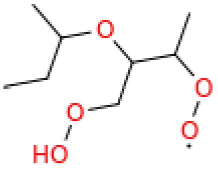
SC8O-DO2H-CO2	CCC(C)OC(COO)CCO[O]	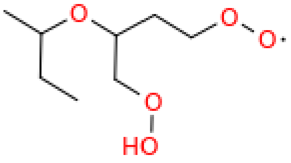

SC8O-DO2H-1O2	CC[C@@](C)(O[O])OC(COO)CC	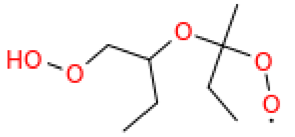
SC8O-CO2H-AO2	CCC(C)O[C@@](C)(O[O])CCOO	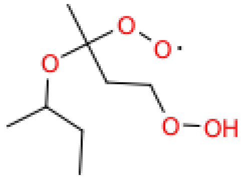
SC8O-CO2H-DO2	CCC(C)OC(CO[O])CCOO	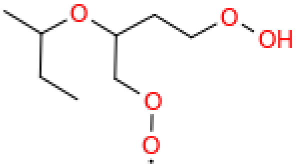
**!KHP**		
SC4OC4KETB-A	CC([C@@](C)(OO)OC(C)CC)=O	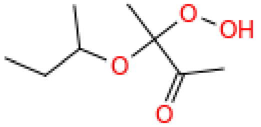
SC4OC4KETB-D	CC(C(COO)OC(C)CC)=O	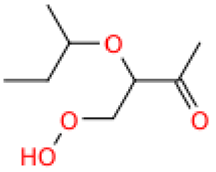
SC4OC4KETB-1	CC(C(C)O[C@@](C)(OO)CC)=O	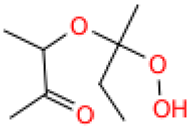
SC4OC4KETC-A	CCC(C)O[C@](C)(OO)CC=O	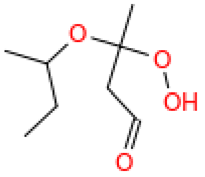
SC4OC4KETC-D	CCC(C)OC(COO)CC=O	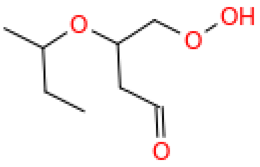
SC4OC4KETD-B	CC(OO)C(C=O)OC(C)CC	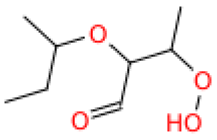

SC4OC4KETD-C	CCC(C)OC(C=O)CCOO	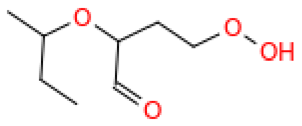
SC4OC4KETD-1	CCC(C=O)O[C@@](C)(OO)CC	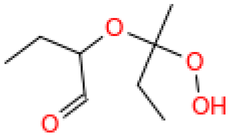
**!P(OOH)2 and its intermediates**		
P(OOH)2AC-D	CCC(C)O[C@@]([CH2])(OO)CCOO	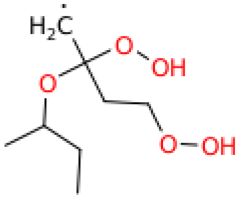
P(OOH)2A1-B	CC[C@@](C)(OO)O[C@@](C)(OO)[C]C	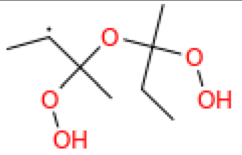
P(OOH)2A2-3	[C]C(OO)C(C)O[C@@](C)(OO)CC	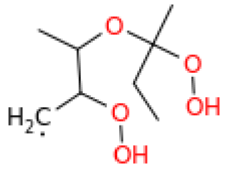
QOOHC-CYCAD	CCC1(CO1)OC(C)CCOO	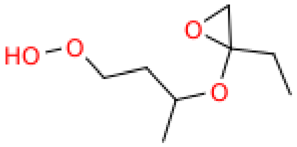
QOOH1-CYCAB	CC1C(C)(O1)O[C@@](C)(OO)CC	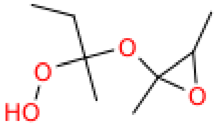
QOOHA-CYC23	CC[C@](C)(OO)OC(C)C1CO1	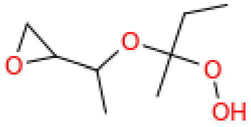
QOOHA-ENE1	C/C=C(C)/O[C@@](C)(OO)CC	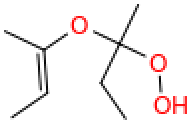
QOOHC-ENED	CCC(C)OC(CCOO)=C	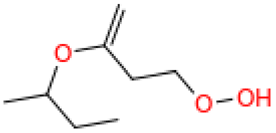

**!intermediates**		
C2H5O2(65)		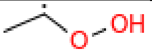
S(900)	C[CH]OO	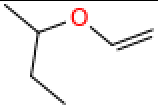
S(1216)	CC=COC(C)CC	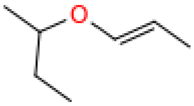
S(478)	C=C(O)C=O	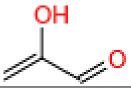
S(7512)	C=C(O)C(C)=O	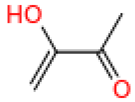
C4H7O(257)	CC=C(C)[O]	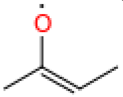
S(7642)	[CH2]C(O)C(C)=O	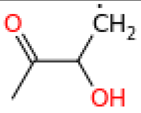
C7H15O	CC(OC(C)[CH2])CC	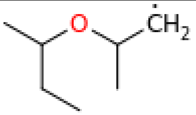
C6H13O	CC(O[CH]C)CC	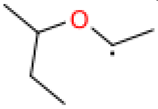
