# Supplementary material for: Chemical Kinetics Investigations of Dibutyl Ether Isomers Oxidation in a Laminar Flow Reactor
Source: Energy Fuels. 2024 Oct 31;38(22):22501–15. doi: 10.1021/acs.energyfuels.4c03432 (PMC11586913; doi:10.1021/acs.energyfuels.4c03432)
Supplement: Supplementary file 7 — ef4c03432_si_007.pdf [file ef4c03432_si_007.pdf]

Chemical kinetics investigations of dibutyl ether isomers oxidation in a laminar flow reactor

Nimal Naser,[†] Samah Y. Mohamed,[†] Gina M. Fioroni,[†] Seonah Kim,[†,‡] and Robert L. McCormick[*,†]

† *National Renewable Energy Laboratory, Golden, CO 80401, USA*
‡ *Chemistry Department, Colorado State University, Fort Collins, CO 80523, USA*

* E-mail: robert.mccormick@nrel.gov

Arrhenius paramters for the β-scission of DIBE radical

High-Pressure-Limit Rate Parameters for DIBE b-scission reaction fit between 400 and 1,500 K. A is the pre-exponential factor, n is the temperature fitting parameter, and E_a is the activation energy in kcal/mol

	Log A	n	Ea (kcal/mol)
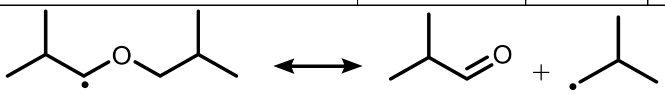			
DIBE-A = CC(C)C=O + [CH2]C(C)C	12.82	0.23	25.31
reverse rate	-23.53	3.26	15.07
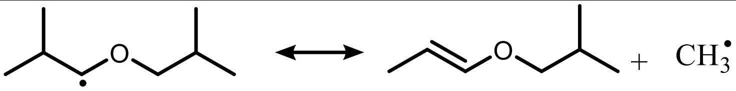			
DIBE-A = C/C=C/OCC(C)C + [CH3]	14.17	0.13	33.60
reverse rate	-17.13	2.02	5.74
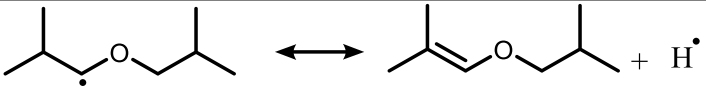			
DIBE-A = C/C(C)=C/OCC(C)C + [H]	12.29	0.35	36.20
reverse rate	-12.17	0.77	-0.59
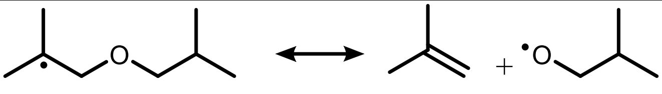			
DIBE-B = CC(C)=C + [O]CC(C)C	11.31	0.65	23.60
reverse rate	-20.23	2.66	2.04

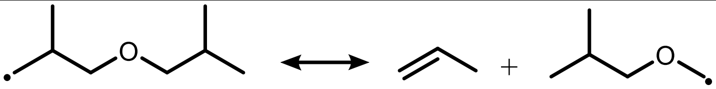			
DIBE-C = C=CC + CC(C)CO[CH2]	11.87	0.53	28.19
reverse rate	-21.18	3.05	5.73
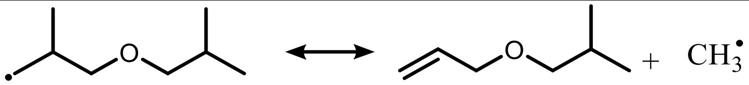			
DIBE-C = C=CCOCC(C)C + [CH3]	11.79	0.56	32.50
reverse rate	-20.51	2.70	5.13
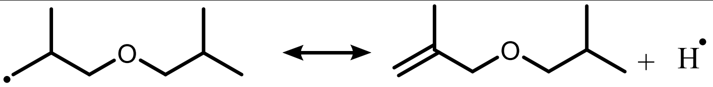			
DIBE-C = C=C(C)COCC(C)C + [H]	9.97	0.87	35.31
reverse rate	-14.22	1.50	1.19
